# An improved method for circular RNA purification using RNase R that efficiently removes linear RNAs containing G-quadruplexes or structured 3′ ends

**DOI:** 10.1093/nar/gkz576

**Published:** 2019-07-03

**Authors:** Mei-Sheng Xiao, Jeremy E Wilusz

**Affiliations:** Department of Biochemistry and Biophysics, University of Pennsylvania Perelman School of Medicine, Philadelphia, PA 19104, USA

## Abstract

Thousands of eukaryotic protein-coding genes generate circular RNAs that have covalently linked ends and are resistant to degradation by exonucleases. To prove their circularity as well as biochemically enrich these transcripts, it has become standard in the field to use the 3′-5′ exonuclease RNase R. Here, we demonstrate that standard protocols involving RNase R can fail to digest >20% of all highly expressed linear RNAs, but these shortcomings can largely be overcome. RNAs with highly structured 3′ ends, including snRNAs and histone mRNAs, are naturally resistant to RNase R, but can be efficiently degraded once a poly(A) tail has been added to their ends. In addition, RNase R stalls in the body of many polyadenylated mRNAs, especially at G-rich sequences that have been previously annotated as G-quadruplex (G4) structures. Upon replacing K^+^ (which stabilizes G4s) with Li^+^ in the reaction buffer, we find that RNase R is now able to proceed through these sequences and fully degrade the mRNAs in their entirety. In total, our results provide important improvements to the current methods used to isolate circular RNAs as well as a way to reveal RNA structures that may naturally inhibit degradation by cellular exonucleases.

## INTRODUCTION

Most eukaryotic genes contain intronic sequences that must be removed from nascent pre-messenger RNAs by the splicing machinery. This process is highly regulated and can be exploited to allow the generation of a diverse set of mature transcripts from a given gene (reviewed in 1–3). In most cases, introns are spliced out in a sequential order to yield a functional linear mRNA that is exported to the cytoplasm for translation, while the intron lariats are rapidly debranched and degraded in the nucleus. Nevertheless, some intron lariats fail to be debranched and instead accumulate as circular intronic RNAs (ciRNAs) or stable intronic sequence RNAs (sisRNAs) (4,5). In addition, several non-canonical forms of pre-mRNA splicing events have been identified, including trans-splicing reactions that generate chimeric RNAs by joining together regions from separate pre-mRNAs (reviewed in 6). The splicing machinery can also ‘backsplice’ to join a splice donor to an upstream splice acceptor (e.g. join the end of exon 2 to the beginning of exon 2), thereby generating an exonic circular RNA (circRNA) with covalently linked ends (reviewed in 7–10). Backsplicing is often facilitated by intronic complementary sequences that base pair to one another and bring the intervening splice sites close together (11–13). These reactions are further controlled by RNA binding proteins (14–18), exon skipping events (19) and by the levels of core spliceosomal components (20).

Some exonic circular RNAs are expressed at much higher levels than their associated linear mRNAs, especially in neuronal tissues (21–23). These transcripts have been proposed to bind and sequester microRNAs or RNA binding proteins or, alternatively, act as templates for translation (14,24–28). However, most backsplicing reactions are far less efficient than canonical splicing (29) and thus the vast majority of circular RNAs are expressed at very low levels. These transcripts can be detected in RNA-seq datasets by searching for reads that span backsplicing junctions, but significant experimental and computational issues limit the accuracy of current circular RNA annotations (reviewed in 30). Most notably, reverse transcription can introduce template-switching artifacts (31,32) and there is surprisingly little overlap in the circular RNAs predicted by different computational pipelines (33,34).

It thus has become common to validate putative circular RNAs by confirming that they are resistant to degradation by the highly processive 3′-5′ exonuclease Ribonuclease R (RNase R) (22). RNase R has helicase activity that enables the enzyme to degrade highly structured RNAs, including ribosomal RNAs, provided that a single-stranded 3′ overhang is present (35–37). Bona fide exonic and intronic circular RNAs should become enriched after RNase R treatment (as linear RNAs are depleted from the population), thereby confirming their covalently closed structure and making their detection easier. Nevertheless, it is known that some abundant cellular RNAs with highly structured ends, such as small nuclear RNAs (snRNAs) and tRNAs, are poor substrates for RNase R (38).

Here, we analyzed RNA-seq data from human HeLa cells to systematically characterize the breadth of linear and circular RNAs that are resistant to degradation by RNase R. As expected, thousands of RNase R-resistant circular RNAs derived from exons and introns were identified, but we also found many linear RNAs that fail to be degraded by RNase R. Some of these linear RNAs, including histone mRNAs and several classes of small noncoding RNAs, are naturally resistant to RNase R as they lack a sufficiently long single-stranded 3′ overhang. Surprisingly, we also found that RNase R was unable to fully digest hundreds of polyadenylated protein-coding mRNAs as the enzyme stalls within these transcripts at G-quadruplex (G4) structures. Upon replacing K^+^ (which stabilizes G4s) with Li^+^ in the reaction buffer, RNase R was able to proceed through these sequences and fully degrade the mRNAs. By combining A-Tailing and RNase R digestion in the presence of Li^+^, we show that a more pure population of circular RNAs can be isolated.

## MATERIALS AND METHODS

### HeLa cell culture and transfections

HeLa cells were grown at 37°C and 5% CO_2_ in Dulbecco's modified Eagle's medium (DMEM) containing high glucose (Thermo Fisher Scientific 25030081), supplemented with 10% fetal bovine serum and 1% (v/v) penicillin-streptomycin. For transfections, 4 × 10^5^ cells were seeded in six-well plates and 1 μg of plasmid was transfected using 5 μl Lipofectamine 2000 (Thermo Fisher Scientific 11668019). After 24 h, total RNA was isolated using Trizol (Thermo Fisher Scientific 15596018) as per the manufacturer's instructions.

### RNA-seq library preparation and analysis

10 μg of HeLa total RNA from two biological replicates was incubated at 37°C for 15 min in 20 μl reactions with KCl-containing buffer (final concentrations: 20 mM Tris–HCl (pH 8.0), 0.1 mM MgCl_2_, and 100 mM KCl) -/+ 10 U RNase R (Lucigen RNR07250). The RNAs were then purified using the RNeasy MinElute Cleanup Kit (Qiagen 74204) following the manufacturer's instructions and eluted in 30 μl nuclease-free water. 500 ng of each RNA sample was used as starting material for RNA-seq library preparation using the TruSeq Stranded Total RNA Library Prep Gold kit (Illumina 20020598) following the manufacturer's instructions except that a 1/100 dilution of the ERCC Spike-In Mix (Thermo Fisher Scientific 4456740) was added to each sample after ribosomal RNA depletion. Libraries were sequenced from a single end for 150 nt on the Illumina NextSeq 500 platform using TG NextSeq 500/550 High Output Kit v2 (Illumina TG-160–2002).

Sequencing reads were mapped to the human genome (hg38, downloaded from UCSC) using TopHat2 (version 2.0.14) with default parameters (39). For each library, read counts were calculated per gene in a strand-specific manner and Cufflinks version 2.2.1 (40) was used to estimate transcript levels (RPKM, Reads Per Kilobase per Million) based on Ensembl gene annotations (hg38, version 90). The RPKM ratio of RNase R/Control was then calculated for each of the top 25% highly expressed genes. TopHat2 and Cufflinks were similarly used to quantify the levels of ERCC Spike-In sequences in each library. RNA-seq data from Gao *et al.* 2015 (NCBI SRA accession numbers SRR1637089 and SRR1636985) and Jeck *et al.* 2013 (NCBI SRA accession numbers SRR444655 and SRR444974) were downloaded and analyzed in the same way (22,41).

To identify circular RNAs using CIRI2 (version 2.0.6) (41), RNA-seq reads were first mapped to hg38 with bwa (42) and then the alignments were inputted to CIRI2 with default parameters. To identify circular RNAs and ciRNAs using CIRCexplorer2 (43), RNA-seq reads that could not be aligned to hg38 with TopHat2 (version 2.0.14) were used to identify fusion junctions with TopHat2-Fusion (version 2.0.14) (39), which were then inputted to CIRCexplorer2. For transcripts identified by CIRI2 or CIRCexplorer2, at least two junction reads in one of the libraries was required for a circular RNA or ciRNA to be retained for further analysis. Polyadenylation site annotations were downloaded from the PolyA site database (http://exon.umdnj.edu/polya_db/) and the coordinates were liftOvered to hg38.

### Poly(A) tailing and RNase R treatments

10 μg of HeLa total RNA was subjected to poly(A) tailing in a 50 μl reaction using the Poly(A) Tailing Kit (Thermo Fisher Scientific AM1350) following the manufacturer's instructions except that reactions were incubated at 37°C for 10 min and 40 U RNase Inhibitor (Thermo Fisher Scientific N8080119) was added to each reaction. Control reactions were set up similarly except that *Escherichia coli* Poly(A) Polymerase I was omitted. After incubation, RNAs were acid-phenol chloroform extracted, chloroform extracted, ethanol precipitated, and dissolved in nuclease-free water. The RNAs were then incubated at 37°C for the indicated amounts of time in 20 μl reactions that contained 2 μl 10× RNase R Buffer (0.2 M Tris–HCl (pH 8.0), 1 mM MgCl_2_ and 1 M KCl, NaCl or LiCl) and 0.5 μl (10 U) RNase R. Control reactions were similarly performed but water was added in place of the RNase R enzyme. Reactions were purified with RNA Clean & Concentrator-25 (Zymo Research R1018) according to the manufacturer's instructions and the RNA was eluted in 25 μl nuclease-free water. RNA-seq libraries from 500 ng were then generated and analyzed as above using HeLa total RNA from three biological replicates that had been subjected to poly(A) tailing and RNase R treatment (LiCl-containing buffer for 90 min) or the buffer treatments alone.

### RT-qPCR

300 ng of RNA was reverse transcribed using random hexamers and SuperScript III (Thermo Fisher Scientific 18080051) according to the manufacturer's instructions. The cDNA was then diluted 5-fold with H_2_O and RT-qPCR was performed using Power SYBR Green PCR Master Mix (Thermo Fisher Scientific 4367659). RT-qPCR reactions were performed in 15 μl and contained 1 μl of diluted cDNA, 7.5 μl 2× Power SYBR Green PCR Master Mix, 6 μl 1.5 μM gene-specific primer pairs, and 0.5 μl H_2_O. All RT-qPCR primer sequences are provided in Supplementary Table S1. Using the LightCycler 96 Real-Time PCR System (Roche), the following cycling conditions were used: 95°C for 10 min, 40 amplification cycles of 95°C for 15 s followed by 60°C for 1 min, and a final melting cycle of 95°C for 10 s, 65°C for 1 min, and 97°C for 1 s. Relative transcript levels were calculated using the 2^−ΔΔCT^ method. RT-qPCR reactions were performed using three independent biological replicates, with each replicate having two technical replicates.

### Northern blotting

Northern blots using NorthernMax reagents (ThermoFisher Scientific) and oligonucleotide probes were performed as described previously (44). All probe sequences are provided in Supplementary Table S1. Blots were viewed with the Typhoon 9500 scanner (GE Healthcare). Representative blots are shown.

### Ligation-mediated 3′ RACE

10 μg of HeLa total RNA was incubated at 37°C for 15 min in reactions containing 10 U RNase R (Lucigen RNR07250) and KCl-containing buffer followed by RNA purification. 1.5 μg RNA was ligated to microRNA Linker 3 (5′-rAppTTTAACCGCGAATTCCAG/3ddC/) using T4 RNA Ligase I (NEB M0242) and incubated at room temperature for 2 h. The reaction was acid-phenol:chloroform extracted (Thermo Fisher Scientific AM9720) and ethanol precipitated. Reverse transcription was performed using Superscript III (Thermo Fisher Scientific 18080051) and an oligo complementary to the linker (5′-GACTAGCTGGAATTCGCGGTTAAA). 2 μl of cDNA was used as a template for PCR using a gene-specific forward primer (sequences in Supplementary Table S1) and the RT primer. The resultant PCR products were inserted into pGEM-T Easy (Promega A1360) and sequenced.

### Prediction and characterization of RNase R stalling sites

RNA-seq data from all four samples that were incubated in the KCl-containing buffer (Control Rep 1 and 2, RNase R Rep 1 and 2) were inputted to DaPars (45) to estimate RNase R stalling sites. Sites with FDR ≤0.05 that were present in genes with an RPKM ratio (RNase R/Control) ≥1 in both replicates were retained for further analysis only if they also passed manual inspection (located within ∼200 nt of where the RNA-seq signal clearly dropped off after RNase R treatment). A 400 nt window centered on each stalling site was extracted using bedtools (46) and the nucleotide frequency in each window was calculated. As a control, predicted stalling sites were shuffled 100 times in the last exons of highly expressed (top 25%) genes and the nucleotide frequencies of these genomic windows were similarly calculated. To compare the locations of RNase R stalling sites predicted by DaPars to previously annotated G-quadruplexes, the coordinates of G-quadruplexes in RNAs isolated from HeLa cells were obtained (47,48). For each stalling site, the closest G-quadruplex was identified using bedtools.

For each 400 nt window centered on the stalling site predicted by DaPars, the Quadruplex forming G-rich sequences (QGRS) value was calculated (49). When multiple QGRS values were calculated for a window, only the highest value was retained. As a control, the stalling site windows were shuffled once in the last exons of highly expressed (top 25%) genes and QGRS values were similarly calculated.

### Expression plasmid construction

To generate expression plasmids for transfection, G4-containing regions from PPP1R8, SH3BP5, or EML2 were inserted into the 3′ UTR of pcDNA3.1(+) eGFP, which was generated by subcloning mEGFP from mEGFP-N1 (Addgene plasmid #54767) into pcDNA3.1(+). Additional details and the sequences of the inserts for all plasmids are in the Supplementary Material.

### Statistical analyses

For RT-qPCRs, statistical significance for comparisons of means was assessed by Student's *t* test. To calculate a *P*-value for enrichment of G4s near predicted RNase R stalling sites, the Fisher's exact test was used. Mann–Whitney U-test was used to compare the statistical difference of QGRS values, RPKM ratios and ratios of circRNA junction reads.

## RESULTS

### Hundreds of linear RNAs are resistant to digestion by the 3′-5′ exonuclease RNase R

**Figure 1.**
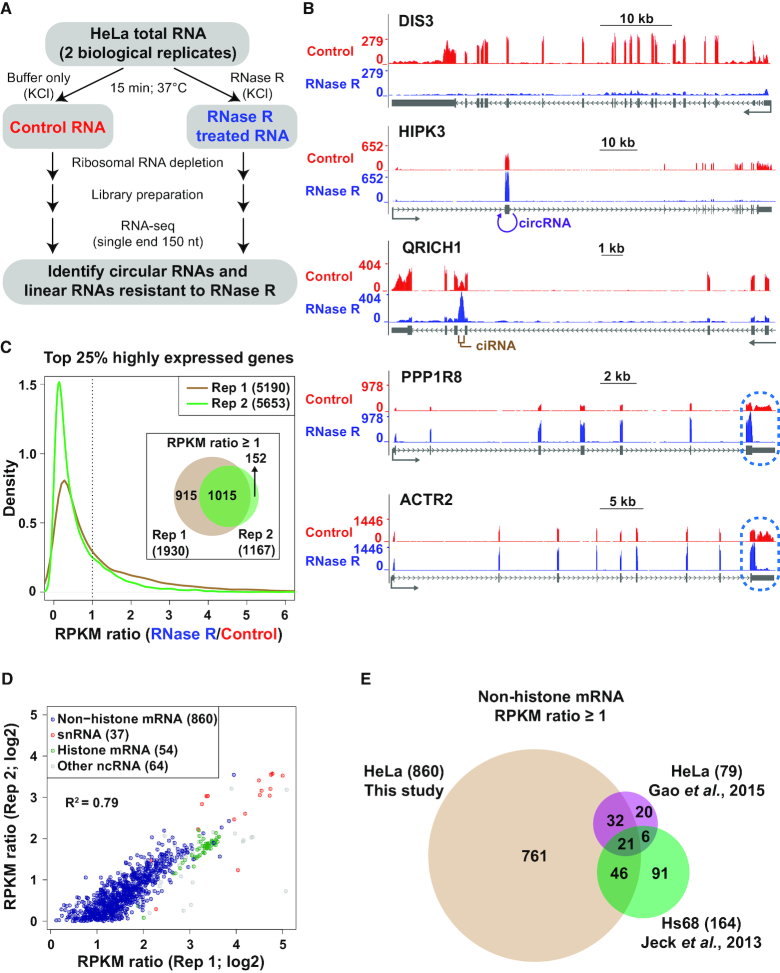
Hundreds of linear RNAs are resistant to RNase R digestion. (**A**) HeLa total RNA was incubated in KCl-containing buffer -/+ RNase R followed by preparation of RNA-seq libraries. (**B**) RNA-seq data generated from control (red) or RNase R treated samples (blue) were used to produce a normalized coverage value over individual nucleotides (Reads per Kilobase per Million [RPKM]). The DIS3, HIPK3, QRICH1, PPP1R8 and ACTR2 loci are shown. Gray arrows below gene models indicate the direction of transcription. Regions that generate an exonic circular RNA (circRNA) or a circular intronic RNA (ciRNA) are denoted in purple and brown, respectively. RNase R stalling in the terminal exons of PPP1R8 and ACTR2 is highlighted in blue. (**C**) For the top 25% of highly expressed genes in each RNA-seq replicate, the RPKM ratio (RNase R/Control) was calculated. A density plot showing the distribution of RPKM ratios is shown along with a Venn diagram denoting the number of genes with a RPKM ratio ≥1 in each replicate. (**D**) Comparison of RPKM ratios for the 1,015 genes that had a RPKM ratio ≥1 in both RNA-seq replicates. Genes encoding non-histone mRNAs (blue), snRNAs (red), histone mRNAs (green), and other noncoding RNAs (gray) are denoted. (**E**) The set of 860 non-histone genes with a RPKM ratio ≥1 in our data was compared to previously published RNA-seq experiments that analyzed RNA expression levels –/+ RNase R treatment. Non-histone mRNA genes with a RPKM ratio (RNase R/Control) ≥1 in each data set are shown.

To globally identify RNAs that are resistant to RNase R digestion, HeLa total RNA was incubated with RNase R at 37°C for 15 min or in the KCl-containing reaction buffer alone as a control (Figure 1A). RNAs remaining after these treatments were purified, depleted of ribosomal RNAs, and ERCC Spike-In transcripts were added. RNA-seq libraries from two biological replicates were then prepared, yielding ∼30–55 million reads that uniquely mapped to the human genome (hg38) (Supplementary Figure S1A). The measured RPKM values of the ERCC Spike-In transcripts were highly correlated across the RNA-seq libraries, confirming the quality of the preparations (Supplementary Figure S1B and C).

As expected, many linear mRNAs were efficiently depleted by RNase R treatment, including from the DIS3, HIPK3 and QRICH1 gene loci (Figure 1B). In contrast, exonic circular RNAs (circRNAs), such as the one derived from backsplicing of exon 2 of HIPK3 (11), and circular intronic RNAs (ciRNAs), including the one derived from QRICH1 (4), became enriched after RNase R treatment (Figure 1B). Using the algorithms CIRI2 (41) and CIRCexplorer2 (43), thousands of exonic circRNAs (with at least 2 independent backsplicing junction-spanning reads) were identified in each library (Supplementary Figure S2A and Table S2). 725 exonic circRNAs were identified by both algorithms in both biological replicates from control treated RNAs, whereas this number increased to 4540 after RNase R treatment (Supplementary Figure S2B), 80.8% of which are currently annotated in circBase (50). Accordingly, circRNA junction reads were overall more abundant (3.9–6.8-fold) after RNase R treatment (Supplementary Figure S2C). Circular intronic RNAs were similarly identified using CIRCexplorer2 (Supplementary Figure S2D-F and Supplementary Table S3). In general, ciRNAs were found to be much less abundant than exonic circRNAs, with only 6 detected in both biological replicates from control treated RNAs and 130 detected after RNase R treatment (Supplementary Figure S2E).

Upon examining the sequencing data further, we noted a number of additional transcripts that appeared to be resistant to RNase R treatment, including from the PPP1R8 and ACTR2 gene loci (Figure 1B). RNA-seq signal from these loci remained in all exons after RNase R treatment, including in the first and last exons that should not be able to give rise to circular RNAs due to the presence of a 5′ cap and a poly(A) tail, respectively. We thus reasoned that the PPP1R8 and ACTR2 linear mRNAs may be naturally resistant to RNase R, although it should be noted that some digestion was observed from their 3′ ends (discussed in detail below). To globally determine the number of gene loci that generate RNAs resistant to RNase R, we determined the sequencing coverage (RPKM) for the top 25% of highly expressed genes in control and RNase R treated samples and then calculated the RPKM ratio (RNase R/Control) of each gene (Figure 1C and Supplementary Table S4). Consistent with RNase R efficiently degrading most linear mRNAs, the RPKM ratio for most genes was less than 1 (note that lower RPKM ratios were generally observed in Replicate 2, suggesting that RNase R degradation was more efficient in that biological replicate). However, 1,015 genes (representing ∼20% of highly expressed genes) had a RPKM ratio ≥1 in both biological replicates (Figure 1C) and the calculated RPKM ratios were well correlated between the biological replicates (*R*^2^ = 0.79) (Figure 1D). This set of RNase R resistant genes included 860 non-histone protein-coding genes (including PPP1R8 and ACTR2, Figure 1B), 37 small nuclear RNAs (snRNAs), 54 histone-encoding genes, and 64 genes encoding various noncoding RNAs (ncRNAs) (Figure 1D).

Highly abundant exonic circular RNAs can cause the overall gene-level RPKM measurements to remain high after RNase R treatment, as is the case with the RNF13 gene that generates many circular RNAs. However, no exonic circular RNAs were detected from the majority (∼61%) of RNase R resistant protein-coding gene loci (Supplementary Figure S2G). This suggests that linear RNAs generated from these loci have features that cause them to be resistant to RNase R.

To verify that these transcripts are indeed resistant to RNase R, we analyzed two previously published RNA-seq datasets that compared transcript levels –/+ RNase R treatment (22,41). Jeck *et al.* incubated RNA from Hs68 human fibroblasts with RNase R at 40°C for 1 hr (22), whereas Gao *et al.* incubated RNA from HeLa cells with RNase R at 37°C for 1 h (41). 164 and 79 highly expressed non-histone protein-coding genes were found to be resistant to RNase R (RPKM ratio ≥ 1) in these studies (Figure 1E and Supplementary Table S4). A set of 21 genes, including ACTR2 (Figure 1B) and UBE2E1, were resistant to RNase R in all three independent datasets (Supplementary Figure S3). Compared to our study, fewer RNase R resistant genes were identified in these other datasets (likely due to differences in experimental procedures) (Figure 1E), but we nonetheless conclude that a consistent set of RNAs cannot be efficiently degraded by RNase R.

### Addition of A tails *in vitro* enables RNAs with structured 3′ ends to be degraded by RNase R

**Figure 2.**
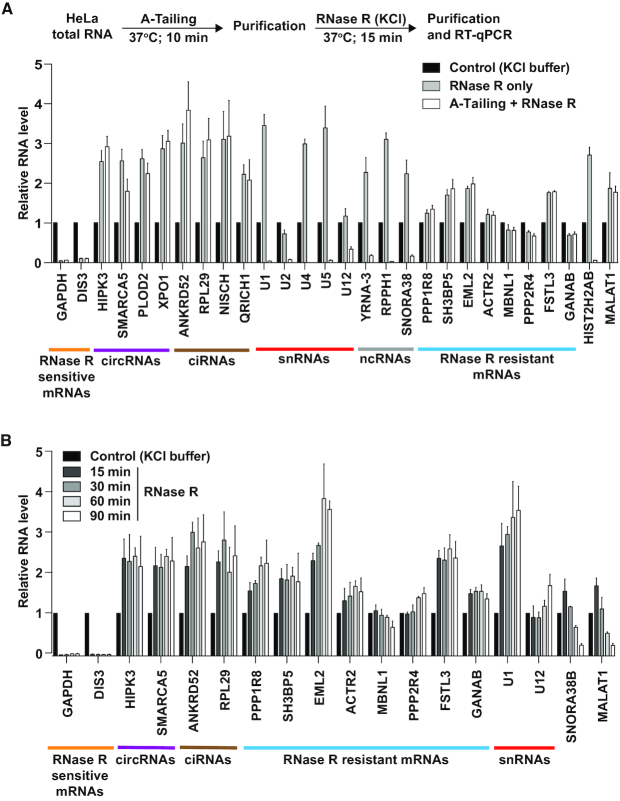
Many mRNAs remain resistant to RNase R digestion after A-Tailing or longer incubation times. (**A**) To determine if addition of single-stranded poly(A) tails enables more efficient digestion by RNase R, purified HeLa total RNA was incubated with E-PAP prior to RNase R digestion (white bars). As controls, RNA was incubated only in the reaction buffers (black bars) or subjected to RNase R digestion alone (gray bars). 300 ng of the remaining RNA was used for reverse transcription followed by qPCR to measure the relative abundances of the indicated transcripts. RNase R sensitive mRNAs (orange), circRNAs (purple), ciRNAs (brown), snRNAs (red), ncRNAs (gray), and RNase R resistant mRNAs (blue) are noted. (**B**) HeLa total RNA was treated at 37°C with buffer (containing KCl) only or RNase R for the indicated amounts of time. 300 ng of the remaining RNA was then used for reverse transcription followed by qPCR to measure the relative abundances of the indicated transcripts. All data were normalized to the control samples and are shown as mean ± SD, *n* = 3.

RNase R can degrade RNAs with extensive secondary structure, including ribosomal RNAs, provided that a single-stranded 3′ overhang of at least seven nucleotides is present (35,36). Optimum binding and activity of RNase R is achieved when the overhang is 10 or more nucleotides in length, and it is thus well established that RNase R is unable to degrade highly structured RNAs such as snRNAs. Accordingly, we noted that a number of RNAs that are known to lack 3′ overhangs were found to be resistant to RNase R in our RNA-seq analysis (Figure 1D and Supplementary Table S4) and subsequent RT-qPCR validation efforts (Figure 2A, gray bars). This included many snRNAs, snoRNAs (reviewed in 51), Y RNAs (reviewed in 52), histone mRNAs (reviewed in 53), and the long noncoding RNA MALAT1 (54–56).

Drawing inspiration from the RPAD circular RNA enrichment method (38), we reasoned that addition of a 3′ terminal poly(A) tail *in vitro* should enable these transcripts to now be susceptible to RNase R digestion (Figure 2A). Indeed, treatment of HeLa total RNA with *E. coli* Poly(A) Polymerase I (E-PAP) prior to incubation with RNase R enabled histone mRNAs (HIST2H2AB), snRNAs (U1, U2, U4, U5 and U12), and other small noncoding RNAs (YRNA-3, RPPH1, SNORA38) to be efficiently degraded by RNase R (Figure 2A, white bars). As expected, exonic circRNAs and ciRNAs remained resistant to RNase R treatment regardless of whether an A-Tailing step was included. However, a number of mRNAs (including PPP1R8 and SH3BP5) also remained resistant to RNase R (Figure 2A), suggesting that features unrelated to the length of the 3′ terminal poly(A) tails can cause RNase R to be unable to fully digest a transcript.

We then tested whether extending the reaction incubation time was sufficient to enable digestion of these mRNAs by RNase R. Even after 90 min, these mRNAs remained resistant to RNase R, and some (e.g. PPP1R8) became increasingly enriched over time (Figure 2B). Extending the reaction time was likewise not sufficient to cause degradation of snRNAs (as no A-Tailing step was employed here), whereas a snoRNA (SNORA38B) and the MALAT1 long noncoding RNA were increasingly digested over time. This suggests that the structures at the ends of snoRNAs and MALAT1 may be more dynamic *in vitro*, enabling RNase R engagement and activity.

### RNase R can abruptly stall within mRNAs

**Figure 3.**
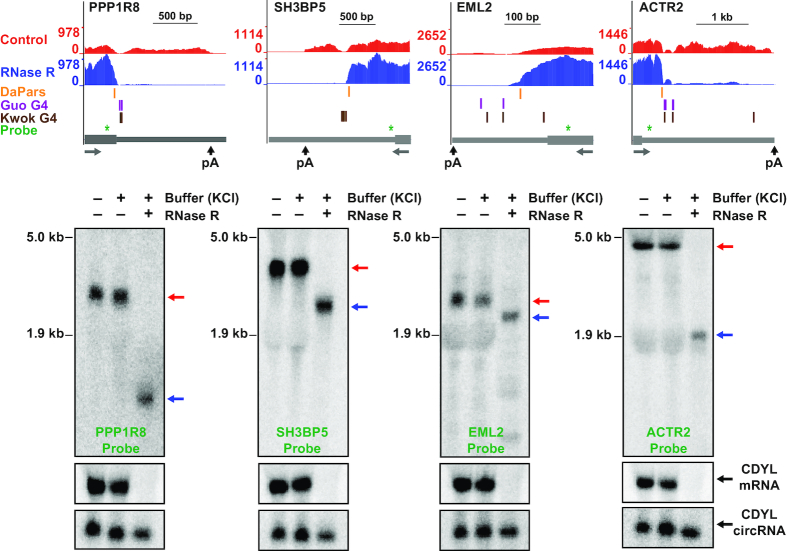
RNase R stalls within the body of many mRNAs. (Top) UCSC genome browser tracks depicting the last exons of four genes that fail to be completely digested by RNase R. Gray arrows below gene models indicate the direction of transcription. RNA-seq data generated from control (red) or RNase R treated samples (blue) are shown along with RNase R stalling sites predicted by DaPars (orange), G-quadruplexes annotated by Guo and Bartel (purple) or Kwok *et al.* (brown), and the northern blot probes (green). (Bottom) HeLa total RNA was treated for 15 min at 37°C with either buffer (containing KCl) only or RNase R and then subjected to Northern blotting. Red and blue arrows indicate full length and partially digested transcripts, respectively. Linear and circular CDYL transcripts were used to confirm RNase R activity.

To understand why mRNAs like PPP1R8 and ACTR2 are resistant to RNase R digestion, we looked further at the RNA-seq data and noted that these transcripts appeared to be truncated at their 3′ ends after RNase R treatment (Figures 1B and 3, top). This suggested that either (i) RNase R was able to remove hundreds of nucleotides from the mRNA 3′ ends before abruptly stalling or (ii) multiple transcripts are produced from these genes, and only some of them are resistant to RNase R. To distinguish between these models, we treated total RNA with RNase R and then performed Northern blots (Figure 3, bottom). A singular prominent transcript was generated from the PPP1R8 and ACTR2 genes, and its expression was unaffected by treatment with the KCl-containing buffer. In contrast, RNase R treatment converted these transcripts into a faster migrating species, consistent with RNase R being able to degrade from the 3′ end of the linear mRNA until it abruptly stalled. Similar stalling patterns were observed on Northern blots probing the SH3BP5 and EML2 mRNAs (Figure 3). To confirm the locations of these RNase R stalling sites, ligation-mediated 3′ RACE was performed. After RNase R treatment in the KCl-containing buffer, the 3′ ends of these mRNAs clustered near where the RNA-seq signal dropped off (Supplementary Figure S4), consistent with the Northern blotting data (Figure 3). This suggested the existence of sequences and/or structures at these mRNA regions that prevented further digestion by RNase R.

### RNase R stalling regions are G-rich and can form G-quadruplexes

**Figure 4.**
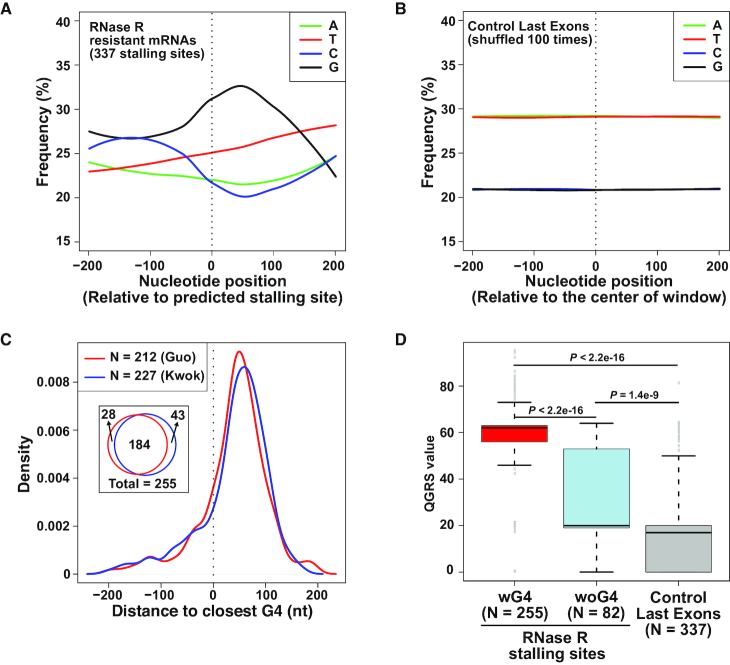
RNase R often stalls at G-rich regions that form G-quadruplexes. (**A**) Average nucleotide frequencies in the regions flanking the 337 RNase R stalling sites that were predicted by DaPars. Location of stalling site is denoted by dashed vertical line. (**B**) Stalling site locations were shuffled 100 times in the last exons of highly expressed (top 25%) genes and the average nucleotide frequencies were calculated as in A. (**C**) The locations of the 337 stalling sites predicted by DaPars were compared to annotated G-quadruplexes defined by Guo and Bartel (red) or Kwok *et al.* (blue). The density plot shows the distribution of distances from the predicted stalling sites to the nearest G-quadruplex in each dataset. The Venn diagram denotes the number of stalling sites with an annotated G-quadruplex within ±200 nt. (**D**) Quadruplex forming G-rich sequences (QGRS) values were calculated for the 400 nt regions flanking the 255 stalling sites with an experimentally annotated G4 nearby (red), the 82 stalling sites without an experimentally annotated G4 nearby (blue), and 337 shuffled stalling sites (gray). Box plots show the 25th–75th percentiles and whiskers represent extreme data points no more than 1.5 times the interquartile range. *P* values were calculated by Mann–Whitney U-test.

To characterize the features present at or near RNase R stalling sites, we first took advantage of the algorithm DaPars (45) to predict stalling sites within the 860 non-histone protein-coding genes that were identified as resistant to RNase R (Figure 1D). Using RNA-seq data as an input, DaPars is able to identify RNA 3′ ends independent of gene model annotations. The algorithm instead simply searches for localized differences in read density and then uses a linear regression model to identify an approximate 3′ end (Figure 3, top; orange). 337 predicted RNase R stalling sites with an FDR ≤0.05 that passed manual inspection (located within ∼200 nt of where the RNA-seq signal clearly dropped off) were retained for further analysis (Supplementary Table S5), including within the PPP1R8, SH3BP5, EML2 and ACTR2 genes (Figure 3, top). We found that the regions flanking these 337 predicted stalling sites are G-rich (Figure 4A), much more so than random sites selected from last exons of highly expressed genes (Figure 4B).

G-rich regions have the potential to form planar structures when four guanine bases interact with each other via hydrogen bonding, and stacking of multiple G-quartets enables formation of a right-handed helical G-quadruplex (G4) structure (reviewed in 57,58). Monovalent cations, such as K^+^, intercalate into the central core of G4 structures, stabilizing hydrogen-bonded tetrads and enhancing base-stacking interactions. In contrast, cations with smaller ionic radius, such as Li^+^, do not readily stabilize G4 structures. G-quadruplexes that form *in vitro* on RNAs from HeLa cells have previously been mapped transcriptome-wide by two groups that exploited the fact that these structures stall reverse transcription in a K^+^-dependent manner (47,48). Interestingly, 255 of the 337 predicted RNase R stalling sites had a nearby G4 (±200 nt from the stalling site) that was experimentally identified in at least one of the two G4 annotation studies (Figure 4C), including in the PPP1R8, SH3BP5, EML2 and ACTR2 mRNAs (Figure 3, top; purple and brown). This overlap between predicted RNase R stalling sites and G4 annotations is highly statistically significant when compared to randomly selected regions in last exons (Supplementary Figure S5). Most of these G-quadruplex structures are located slightly downstream of the predicted RNase R stalling site (Figure 4C), but it is important to keep in mind that DaPars only gives an approximate stalling site. Furthermore, the DaPars predicted site is often slightly upstream of where the RNA-seq signal drops off (Figure 3 and Supplementary Figure S4), suggesting that RNase R likely stalls abruptly upon encountering the G4.

To understand more about the 82 DaPars-predicted RNase R stalling sites without an experimentally identified G4, we used the QGRS Mapper program (49) to predict the presence of G-quadruplex forming sequences ±200 nt from the stalling site. Compared to randomly selected regions in last exons, the regions flanking these 82 RNase R stalling sites have a much higher potential to form G-quadruplexes (Figure 4D). These results suggest that most of the RNase R stalling sites within mRNAs may be due to the presence of G-quadruplex structures.

### Replacing K^+^ with Na^+^ or Li^+^ enables RNase R to digest G-quadruplex containing mRNAs

**Figure 5.**
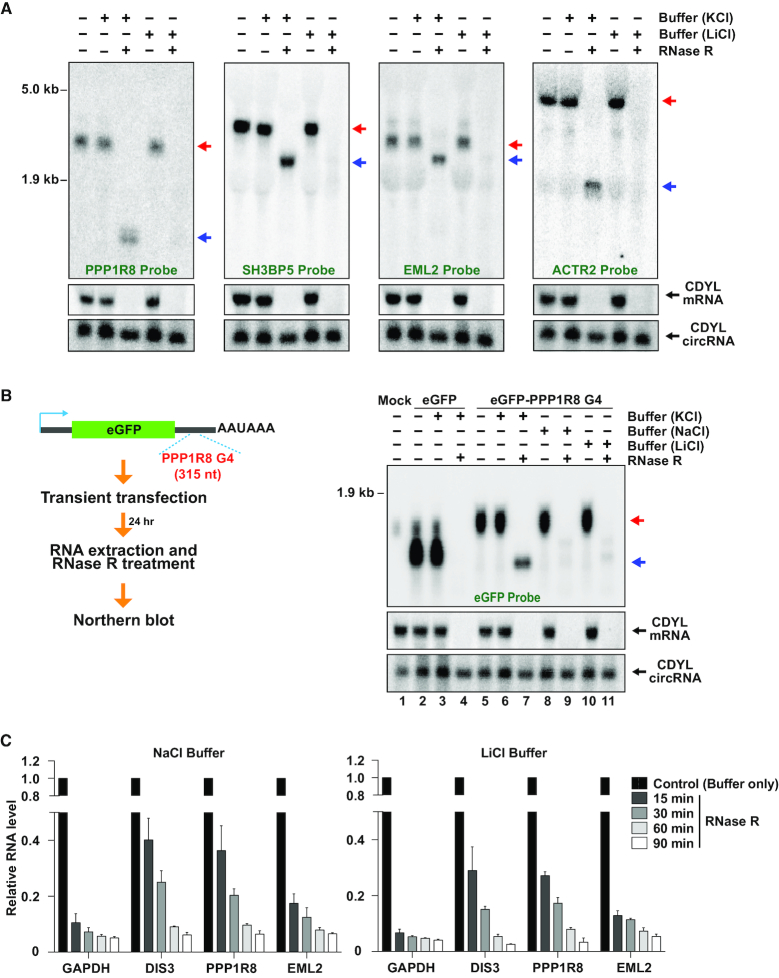
Replacing K^+^ with Na^+^ or Li^+^ in the reaction buffer enables RNase R to fully digest mRNAs containing G-quadruplexes. (**A**) HeLa total RNA was treated for 15 min at 37°C with buffer (containing KCl or LiCl) only or RNase R and then subjected to Northern blotting. Red and blue arrows indicate full length and partially digested transcripts, respectively. Linear and circular CDYL transcripts were used to confirm RNase R activity. (**B**) A 315 nt region containing the PPP1R8 G-quadruplex was inserted into the 3′ UTR downstream of eGFP. Plasmids were transfected into HeLa cells followed by isolation of total RNA, treatment for 15 min at 37°C with RNase R, and analysis by Northern blotting. (**C**) HeLa total RNA was treated at 37°C with buffer (containing NaCl or LiCl) only or RNase R for the indicated amounts of time. 300 ng of the remaining RNA was then used for reverse transcription followed by qPCR to measure the relative abundances of the indicated transcripts.

Given that G-quadruplex structures are stabilized by the presence of K^+^ but not smaller cations (reviewed in 57,58), we reasoned that changing the buffer used in the RNase R reactions from one containing KCl to one containing LiCl or NaCl may diminish G4 formation, thereby allowing more efficient degradation of G4-containing mRNAs. Indeed, when HeLa total RNA was incubated with RNase R in the presence of LiCl rather than KCl, stalling of RNase R was eliminated at the PPP1R8, SH3BP5, EML2 and ACTR2 genes and these mRNAs appeared to be fully degraded (Figure 5A). To further verify these results, we cloned a 315-nt region flanking the PPP1R8 RNase R stalling site into the 3′ UTR of an eGFP expression plasmid (Figure 5B). eGFP mRNA is normally efficiently degraded by RNase R (Figure 5B, lane 4), but inclusion of the PPP1R8 region caused potent stalling of RNase R within the eGFP mRNA when the reactions were performed in the presence of KCl (Figure 5B, lane 7). In contrast, this truncated RNA species was not observed when RNase R digestion reactions were performed in the presence of NaCl (Figure 5B, lane 9) or LiCl (Figure 5B, lane 11). Similar results were obtained when regions flanking the SH3BP5 (Supplementary Figure S6A) or EML2 stalling sites (Supplementary Figure S6B) were cloned into the eGFP 3′ UTR. We thus conclude that K^+^-dependent G-quadruplexes are able to potently block RNase R digestion, but that changing the buffer conditions is able to alter the mRNA structure to allow efficient digestion.

It should be noted that degradation by RNase R is not as efficient in NaCl- or LiCl-containing buffer, and we found that longer incubation times are often required (Figure 5C). For example, expression of the linear DIS3 mRNA was reduced to <10% when RNase R reactions were performed in KCl-containing buffer (Figure 2), but 30–40% of this mRNA still remained after 15 min incubation in NaCl- or LiCl-containing buffer (Figure 5C). Upon longer incubation (e.g. 90 min), <10% of the DIS3 mRNA as well as the G4-containing PPP1R8 and EML2 mRNAs remained after digestion in the NaCl- or LiCl-containing buffer.

### A-Tailing followed by RNase R treatment in LiCl-containing buffer allows more efficient enrichment of circular RNAs

**Figure 6.**
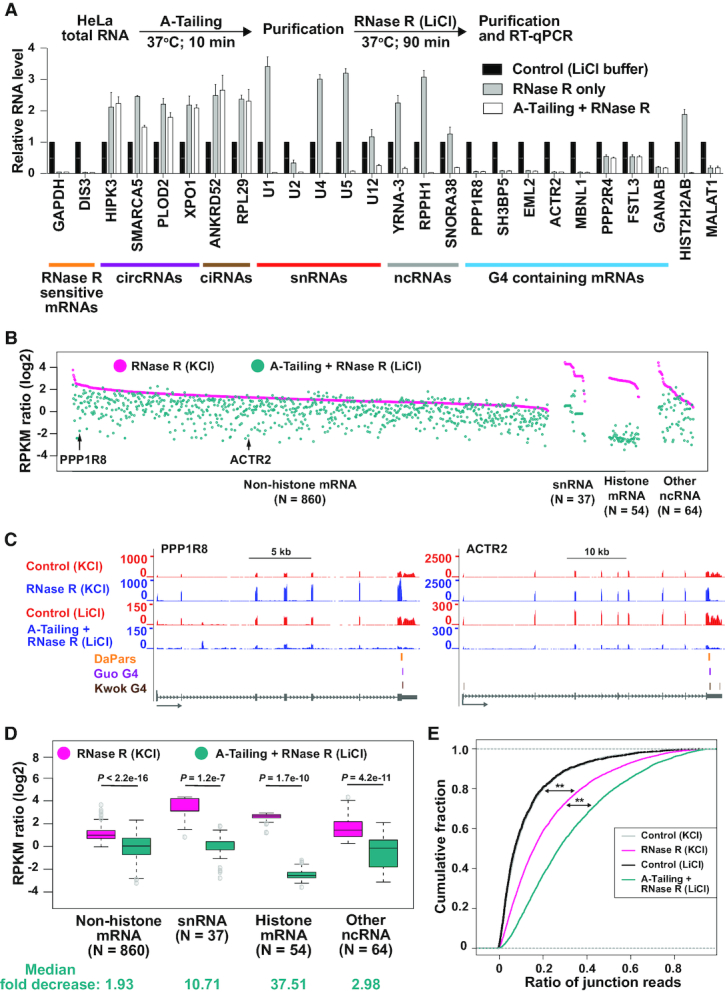
A-Tailing followed by RNase R treatment in LiCl-containing buffer allows more efficient depletion of linear RNAs. (**A**) Purified HeLa total RNA was incubated with E-PAP prior to RNase R digestion in the presence of LiCl buffer (white bars). As controls, RNA was incubated only in the reaction buffers (black bars) or subjected to RNase R digestion alone (gray bars). 300 ng of the remaining RNA was used for reverse transcription followed by qPCR to measure the relative abundances of the indicated transcripts. RNase R sensitive mRNAs (orange), circRNAs (purple), ciRNAs (brown), snRNAs (red), ncRNAs (gray), and RNase R resistant mRNAs (blue) are noted. (**B**) For the 1015 genes that failed to be degraded by RNase R in the presence of KCl (RPKM ratio ≥ 1), the respective RPKM ratio (RNase R/Control) was determined after A-Tailing and RNase R treatment in the presence of LiCl (cyan). Data are grouped according to transcript class and then ranked by the RPKM ratio observed in the KCl dataset (pink). Each dot represents a specific gene, with PPP1R8 and ACTR2 marked by arrows. (**C**) RNA-seq tracks, highlighting the PPP1R8 and ACTR2 loci. Gray arrows below gene models indicate the direction of transcription. RNase R stalling sites predicted by DaPars (orange) and G-quadruplexes annotated by Guo and Bartel (purple) or Kwok *et al.* (brown) are shown. (**D**) Box plots depicting RPKM ratios (RNase R/Control) for non-histone mRNA, snRNA, histone mRNA, and other ncRNA gene loci when RNA was treated with RNase R in the presence of KCl (pink) or subjected to A-Tailing followed by RNase R treatment in the presence of LiCl (cyan). Box plots show the 25th–75th percentiles and whiskers represent extreme data points no more than 1.5 times the interquartile range. Mann–Whitney U-test was used to determine the statistical significance. Median fold decreases for each class were calculated and are denoted at the bottom. (**E**) Cumulative distribution functions of circRNA junction reads ratio (average number of circRNA junction reads predicted by CIRI2/total spliced reads) in RNA-seq datasets generated from the indicated treatment conditions. All genes that produced a circular RNA with at least 2 junction reads in one of the samples are included. Mann–Whitney U-test was used to determine the statistical significance. ^∗∗^*P* < 2.2e–16.

In our RNA-seq analyses, linear transcripts from >1000 genes were resistant to RNase R digestion (Figure 1C, D) and our follow-up analyses indicate this is often due to either (i) the lack of a single-stranded 3′ overhang (Figure 2A) or (ii) the presence of a G-quadruplex structure (Figures 4 and 5). To overcome both of these issues, we reasoned we should first treat total RNA with E-PAP followed by digestion with RNase R in the presence of LiCl (Figure 6A, top). RT-qPCR on a focused set of transcripts revealed that this combination of treatments efficiently removed snRNAs and other noncoding RNAs, G4-containing mRNAs, as well as histone mRNAs, while still allowing exonic circular RNAs and ciRNAs to become enriched (Figure 6A, bottom; white bars). In contrast and as expected, leaving out the A-Tailing step had no effect on depletion of G4-containing mRNAs by RNase R in LiCl-containing buffer, but RNAs with structures ends (e.g. snRNAs, ncRNAs and histone mRNAs) failed to be digested (Figure 6A, gray bars).

To more fully test the efficiency of combining A-Tailing with RNase R digestion in the presence of LiCl-containing buffer, RNA-seq experiments were performed from three biological replicates (Supplementary Figure S7A). ERCC Spike-In transcripts confirmed the high quality of the sequencing data (Supplementary Figure S7B). The sequencing coverage (RPKM) for the top 25% of highly expressed genes was determined in control and A-Tailing + RNase R treated samples, which was used to calculate the RPKM ratio (A-Tailing + RNase R/Control) of each gene (Supplementary Figure S8A). Compared to the previous RNA-seq libraries that were generated from HeLa RNAs treated with RNase R in the presence of KCl, the combination of A-Tailing and RNase R in the presence of LiCl generally resulted in more efficient degradation of linear RNAs (median 20% decrease in RPKM ratio) (Supplementary Figure S8B). We then focused on the 1015 genes that had a RPKM ratio ≥1 in both biological replicates in the original RNA-seq data (Figure 1C). The vast majority of these genes were more efficiently degraded (as shown by a reduced RPKM ratio) by the combination of A-Tailing and RNase R in the presence of LiCl (Figure 6B and Supplementary Table S4), including transcripts derived from the PPP1R8 and ACTR2 loci (Figure 6C). In addition, snRNAs and histone mRNAs were much more efficiently degraded (>10 and >37-fold, respectively) under these conditions (Figure 6B, D).

**Figure 7.**
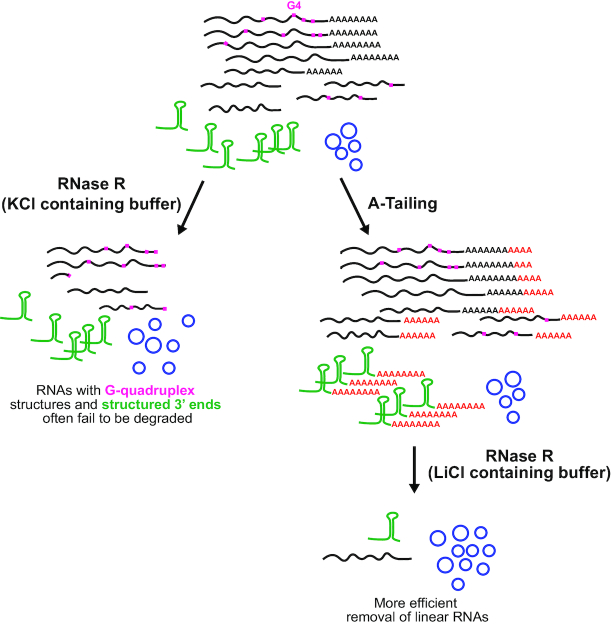
Coupling A-Tailing and RNase R digestion in LiCl-containing buffer enables more efficient enrichment of circular RNAs. (Left) When total RNA is treated with RNase R in KCl-containing buffer, many transcripts with G-quadruplex structures (denoted in pink) or structured 3′ ends (denoted in green) fail to be fully degraded. (Right) Upon inclusion of an A-Tailing step followed by RNase R digestion in LiCl-containing buffer, both of these classes of linear RNAs are more efficiently degraded. This results in isolation of a more pure population of circular RNAs.

Given that linear RNAs that contain internal G-quadruplexes or lack a single-stranded 3′ overhang were more efficiently degraded by this new procedure, we expected to see a greater enrichment of circular RNAs. Indeed, backsplicing junction reads were enriched ∼12-fold (over control treatments) by the combination of A-Tailing + RNase R (Supplementary Figure S9A-C and Supplementary Table S2). This enrichment of exonic circular RNAs is 2–3-fold higher than we previously obtained using RNase R in KCl-containing buffer (Supplementary Figure S2C) and, accordingly, backsplicing junction reads comprised a larger percentage of total spliced reads from circRNA producing genes (Figure 6E). Circular intronic RNA junction reads were likewise enriched to a higher extent by the combination of A-Tailing + RNase R treatment (Supplementary Figure S9D–F and Supplementary Table S3). Together, these results demonstrate that coupling A-Tailing with RNase R in LiCl-containing buffer enables generally more efficient degradation of linear RNAs, thereby allowing higher enrichment of circular RNA transcripts (Figure 7).

## DISCUSSION

Recent work has revealed that thousands of eukaryotic genes generate mature transcripts with covalently linked ends, including exonic circular RNAs and circular intronic RNAs. Some of these transcripts accumulate to high levels, but most are expressed at very low levels (reviewed in 7–10). Given that circular RNAs are, by definition, resistant to decay by exonucleases, it has become common to enrich these transcripts by degrading linear RNAs, most often by using the 3′–5′ exonuclease RNase R that is able to efficiently degrade ribosomal RNAs (22). Nevertheless, RNase R requires a single-stranded 3′ overhang to initiate decay (59), and it was previously shown that RNase R is unable to degrade RNAs with highly structured ends, such as snRNAs and tRNAs (38). In the current study, we further demonstrate that RNase R often stalls at G-quadruplex (G4) structures within the body of many mRNAs. By including an A-Tailing step (analogous to the RPAD method that was developed by (38)) and then replacing K^+^ (which stabilizes G4s) with Li^+^ (which does not stabilize G4s) in the RNase R reaction buffer, these shortcomings of RNase R can be largely overcome. We find that most linear RNAs are now efficiently degraded by RNase R, thereby allowing a more pure population of circular RNAs to be isolated (Figure 7).

G-quadruplex structures have been previously identified in many transcripts using biophysical (e.g. circular dichroism spectroscopy), chemical (e.g. in-line probing), antibody-based, reverse transcriptase-based, and/or computational approaches (reviewed in 57,58). In eukaryotic transcriptomes, sequences that can fold into G4 structures are enriched in 5′ and 3′ UTRs (47,48), and some of these G4s have been suggested to affect key steps in gene expression, including transcription, splicing, polyadenylation, RNA localization, and translation (reviewed in 57,58). Our data confirm that G-quadruplexes readily form *in vitro* in the presence of K^+^ but not Li^+^ cations, and that these structures can cause abrupt stalling of polymerases and RNA decay enzymes. Besides RNase R (discussed further below), G4s stall reverse transcriptase, as shown by focal decreases in the RNA-seq signal at these regions compared to the signal observed in the surrounding regions, e.g. in the PPP1R8 and SH3BP5 3′ UTRs (Figure 3, top). Whether these G4 structures are functional and fold *in vivo* remains unclear, especially since recent data suggest that G4s are globally unfolded in eukaryotic cells (47). All our treatments have been done *in vitro* on purified RNAs and thus do not reveal insights into their *in vivo* folding status. Nevertheless, it will be interesting in the future to see if transient expression of RNase R in eukaryotic cells causes mRNAs to be degraded from their 3′ ends up until their G-quadruplex structures, thereby enabling the easy identification of G4s that naturally form in specific cell types or conditions.

By coupling an A-tailing step with RNase R treatment in the presence of LiCl, we were able to more efficiently remove many linear RNAs than the standard conditions (presence of KCl) that are used for RNase R assays (Figure 6 and Supplementary Figure S8). Nevertheless, some transcripts were less efficiently degraded under our reaction conditions, likely because the RNase R exonuclease activity is less robust in LiCl (Figure 5C). We further found that there is variation in how efficiently G4-containing regions can be degraded by RNase R. For example, the PPP2R4 and FSTL3 mRNAs were less efficiently degraded than the PPP1R8 or ACTR2 mRNAs, despite all of these transcripts having annotated G4 regions (Figure 6B). This may be due to differences in the structural stability of the individual G4-forming regions or the presence of additional sequence/structural elements that block RNase R digestion elsewhere in the transcript.

Besides increasing the time of incubation of the RNase R reaction (Figure 5C), inclusion of additional exonucleases may allow an even purer population of circular RNAs to be isolated. For example, mRNA 5′ cap structures could be removed using tobacco acid pyrophosphatase (TAP) or RNA 5′ pyrophosphohydrolase (RppH), followed by digestion with the 5′–3′ exonuclease Xrn1 that degrades RNAs bearing a 5′ monophosphate (60). Xrn1 is a highly efficient, processive exoribonuclease that acts on the vast majority of RNA substrates, although some viral structures are known to cause abrupt stalling of Xrn1 (61,62). By using both Xrn1 and RNase R, it may be possible to degrade almost all linear RNAs to completion, including RNAs that have modified nucleotides at their 3′ ends. For example, mature U6 snRNA fails to be degraded by our method (Supplementary Table S4), likely because it ends in a 2′–3′ cyclic phosphate (63). In the current manuscript, we have focused on methods to enrich circular RNAs, but it will be equally interesting in the future to use RNase R to identify endogenous RNAs that are naturally resistant at their mature 3′ ends to exonucleases. For example, the long noncoding RNA MALAT1 is known to end in a 3′ terminal triple helix that protects the transcript from degradation (54–56) and, indeed, we found MALAT1 to be semi-resistant to RNase R digestion (Figure 2).

In summary, we systematically characterized the breadth of linear and circular RNAs that are resistant to degradation by RNase R, which revealed that G-quadruplexes often cause potent stalling of the exonuclease. By changing the reaction conditions, we were able to develop a more highly efficient method for enriching circular RNAs. In addition, this method should allow true circular RNAs to be more accurately distinguished from linear RNAs arising from trans-splicing or gene fusions, which can have splicing junctions that resemble backsplicing junctions.

## DATA AVAILABILITY

All RNA-seq datasets generated in this study are available for download from GEO (GSE130905). Data can also be viewed on the UCSC browser using the following link: https://genome.ucsc.edu/s/meishengxiao/hg38_RNaseR_WiluszLab_Upenn.

## Supplementary Material

gkz576_Supplemental_Files
