# Supplementary material for: An improved method for circular RNA purification using RNase R that efficiently removes linear RNAs containing G-quadruplexes or structured 3′ ends
Source: Nucleic Acids Res. 2019 Jul 3;47(16):8755–69. doi: 10.1093/nar/gkz576 (PMC6895279; doi:10.1093/nar/gkz576)

SUPPLEMENTARY FIGURE LEGENDS

Supplementary Figure S1. RNA-seq mapping statistics and library quality control.

(A) RNA-seq data were mapped to the human genome (hg38) using TopHat2 and the mapping statistics are provided. **(B)** Correlation between RNA input abundance and read density output (RPKM) of the ERCC Spike-In transcripts in each RNA-seq library. **(C)** The measured RPKM values of the ERCC Spike-In transcripts were highly correlated across all the RNA-seq libraries. Pearson correlation coefficients (R^2) are given.

Supplementary Figure S2. Identification of exonic circular RNAs (circRNAs) and intronic circular RNAs (ciRNAs).

(A) CIRI2 and CIRCexplorer2 were used to predict exonic circular RNAs in each of the RNA-seq libraries. At least 2 junction reads were required for a circular RNA to be annotated, and the number of circular RNAs in each library that were predicted by both algorithms is provided. **(B)** Overlap of circular RNAs that were predicted by both algorithms in the control and RNase R treated RNA-seq replicates. **(C)** The percentage of circRNA junction reads (identified by CIRI2 or CIRCexplorer2) from all the reads that uniquely mapped to the genome is shown for each RNA-seq library. As expected, circRNA junction reads were enriched after RNase R treatment. **(D)** CIRCexplorer2 was used to predict circular intronic RNAs (ciRNAs) in each of the RNA-seq libraries. **(E)** Overlap of ciRNAs that were predicted in the control and RNase R treated RNA-seq replicates. **(F)** The percentage of ciRNA junction reads from all the reads that uniquely mapped to the genome is shown for each RNA-seq library. **(G)** For each of the 860 genes that encode non-histone mRNAs and were resistant to RNase R in both RNA-seq replicates (RPKM ratio ≥1), the number of circRNA junction reads predicted by CIRI2 (left) and CIRCexplorer2 (right) was calculated. Genes were then organized into 6 clusters based on the number of junction reads.

Supplementary Figure S3. A set of linear mRNAs fails to be fully degraded by RNase R in multiple RNA-seq datasets.

RNA-seq data generated from control (red) or RNase R treated samples (blue) from three independent studies were used to produce a normalized coverage value over individual nucleotides (Reads per Kilobase per Million [RPKM]). The human UBE2E1 and ACTR2 loci are shown. Gray arrows below gene models indicate the direction of transcription. RNase R stalling in the terminal exons is highlighted in blue. RNase R stalling sites predicted by DaPars (orange) and G-quadruplexes annotated by Guo and Bartel (purple) or Kwok et al. (brown) are shown.

Supplementary Figure S4. 3′ RACE confirmed stalling of RNase R in the body of mRNA transcripts.

A ligation-based 3' RACE approach was used to determine the 3' ends of transcripts derived from PPP1R8 **(A)**, SH3BP5 **(B)**, EML2 **(C)**, and ACTR2 **(D)** after digestion by RNase R in the presence of KCl. Gray arrows below gene models indicate the direction of transcription. Each light blue bar represents an individual sequenced 3' RACE clone. In addition to the RNA-seq data generated from control (red) or RNase R treated samples (blue), RNase R stalling sites predicted by DaPars (orange) and G-quadruplexes annotated by Guo and Bartel (purple) or Kwok et al. (brown) are shown.

Supplementary Figure S5. The regions flanking RNase R stalling sites predicted by DaPars are enriched with G-quadruplexes.

To test for statistical enrichment of G-quadruplexes in the regions (±200 nt) flanking stalling sites defined by DaPars, contingency tables were generated and the Fisher's exact test was used to calculate *P*-values. G-quadruplex annotations from Guo and Bartel (left) or Kwok et al. (right) were used. Control stalling sites were generated by shuffling 337 stalling sites 100 times

in the last exons of highly expressed (top 25%) genes and then counting the number of nearby G-quadruplexes (±200 nt). This analysis revealed that G-quadruplexes are statistically enriched near RNase R stalling sites.

Supplementary Figure S6. Replacing K^+ with Na^+ or Li^+ in the reaction buffer enables RNase R to fully digest reporter mRNAs containing G-quadruplexes.

The G-quadruplex containing region derived from SH3BP5 **(A)** and EML2 **(B)** was inserted into the 3' UTR downstream of eGFP. Plasmids were transfected into HeLa cells followed by isolation of total RNA, treatment for 15 min at 37°C with RNase R, and analysis by Northern blotting.

Supplementary Figure S7. RNA-seq mapping statistics and library quality control for samples treated with A-Tailing followed by RNase R in LiCl-containing buffer.

(A) RNA-seq data were mapped to the human genome (hg38) using TopHat2 and the mapping statistics are provided. **(B)** Correlation between RNA input abundance and read density output (RPKM) of the ERCC Spike-In transcripts in each RNA-seq library. Pearson correlation coefficients (R^2) are given.

Supplementary Figure S8. A-Tailing coupled to RNase R digestion in the presence of LiCl generally leads to more efficient depletion of linear RNAs.

(A) For the top 25% of highly expressed genes (N=5,014), the average RPKM ratio (RNase R/Control) was calculated from the RNA-seq data after total RNA was treated with RNase R in KCl-containing buffer (pink) or subjected to A-Tailing followed by RNase R treatment in the presence of LiCl (cyan). Data are ranked by the RPKM ratio observed in the KCl dataset. **(B)** Box plots depicting RPKM ratios for the top 25% of highly expressed genes after using each RNase R treatment method. Box plots show the 25th-75th percentiles and whiskers represent

extreme data points no more than 1.5 times the interquartile range. Mann-Whitney U-test was used to determine the statistical significance. Compared to the standard RNase R treatment in the presence of KCl, a median decrease in RPKM ratio of 20% was observed after combining A-Tailing and RNase R treatment in the presence of LiCl.

Supplementary Figure S9. Identification of exonic circular RNAs (circRNAs) and intronic circular RNAs (ciRNAs) after using A-Tailing coupled to RNase R digestion.

(A) CIRI2 and CIRCexplorer2 were used to predict exonic circular RNAs in each of the RNA-seq libraries. At least 2 junction reads were required for a circular RNA to be annotated, and the number of circular RNAs in each library that were predicted by both algorithms is provided. **(B)** Overlap of circular RNAs that were predicted by both algorithms in the control and A-Tailing + RNase R treated RNA-seq replicates. **(C)** The percentage of circRNA junction reads (identified by CIRI2 or CIRCexplorer2) from all the reads that uniquely mapped to the genome is shown for each RNA-seq library. As expected, circRNA junction reads were enriched after A-Tailing + RNase R treatment. **(D)** CIRCexplorer2 was used to predict circular intronic RNAs (ciRNAs) in each of the RNA-seq libraries. **(E)** Overlap of ciRNAs that were predicted in the control and A-Tailing + RNase R treated RNA-seq replicates. **(F)** The percentage of ciRNA junction reads from all the reads that uniquely mapped to the genome is shown for each RNA-seq library.

SUPPLEMENTARY TABLE LEGENDS

Supplementary Table S1. Oligonucleotide sequences.

The oligonucleotide sequences for RT-qPCR, Northern blots, 3' RACE, and plasmid cloning are provided.

Supplementary Table S2. HeLa circRNAs identified by CIRI2 and CIRCexplorer2.

For each RNA-seq library, exonic circular RNAs (circRNAs) were predicted using CIRI2 or CIRCexplorer2, as indicated. Number of circRNA junction reads are given. Only circRNAs with at least 2 junction reads in one of the samples are listed. For those circRNAs in circBase, the circBase ID is given.

Supplementary Table S3. HeLa ciRNAs identified by CIRCexplorer2.

For each RNA-seq library, circular intronic RNAs (ciRNAs) were predicted using CIRCexplorer2. Number of ciRNA junction reads are given. Only ciRNAs with at least 2 junction reads in one of the samples are listed.

Supplementary Table S4. RPKM ratios (RNase R/Control) for highly expressed genes in each of the RNA-seq libraries.

For the top 25% of highly expressed genes in each RNA-seq library, RPKM values for each gene were calculated using Cufflinks and then the RPKM ratio (RNase R treatment condition/Control) values were calculated.

Supplementary Table S5. RNase R stalling sites predicted by DaPars in the 860 non-histone mRNAs that failed to be efficiently degraded by RNase R.

RNA-seq data from all four samples that were incubated in the KCl-containing buffer (Control Rep 1 and 2, RNase R Rep 1 and 2) were inputted to DaPars (44) to estimate RNase R stalling sites. Only sites with FDR ≤0.05 that were present in the 860 non-histone mRNA genes with an RPKM ratio (RNase R/Control) ≥1 in both replicates are included. In the 400 nt window centered on each stalling site, we determined whether a G4 was experimentally annotated by Guo and Bartel (Column G) or Kwok et al. (Column H) as well as calculated the Quadruplex forming G-rich sequences (QGRS) value (Column I). Manual inspection was further used to verify that predicted stalling sites were located within ~200 nt of where the RNA-seq signal clearly dropped off after RNase R treatment (Column J). Only those 337 sites that passed manual validation were analyzed further.

SUPPLEMENTARY METHODS

All the G-quadruplex containing expression plasmids were generated from **pcDNA3.1(+) eGFP**, which was made by subcloning mEGFP from **mEGFP-N1** (Addgene Plasmid #54767) into **pcDNA3.1(+)**. The full sequence of **pcDNA3.1(+) eGFP** is as follows:

```
GCAAAAAAGGGAATAAGGGCGACACGGAAATGTTGAATACTCATACTCTTCCTTTTTCAATATTATTGAAGCATTTATCAGGGTTATTGTCTCATGA
GCGGATACATATTTGAATGTATTTAGAAAAATAAACAAATAGGGGTTCCGCGCACATTTCCCCGAAAAGTGCCACCTGACGTCGACGGATCGGGAGA
TCTCCCGATCCCCTATGGTGCACTCTCAGTACAATCTGCTCTGATGCCGCATAGTTAAGCCAGTATCTGCTCCCTGCTTGTGTGTTGGAGGTCGCTG
AGTAGTGCGCGAGCAAAATTTAAGCTACAACAAGGCAAGGCTTGACCGACAATTGCATGAAGAATCTGCTTAGGGTTAGGCGTTTTGCGCTGCTTCG
CGATGTACGGGCCAGATATACGCGTTGACATTGATTATTGACTAGTTATTAATAGTAATCAATTACGGGGTCATTAGTTCATAGCCCATATATGGAG
TTCCGCGTTACATAACTTACGGTAAATGGCCCGCCTGGCTGACCGCCCAACGACCCCCGCCCATTGACGTCAATAATGACGTATGTTCCCATAGTAA
CGCCAATAGGGACTTTCCATTGACGTCAATGGGTGGAGTATTTACGGTAAACTGCCCACTTGGCAGTACATCAAGTGTATCATATGCCAAGTACGCC
CCCTATTGACGTCAATGACGGTAAATGGCCCGCCTGGCATTATGCCCAGTACATGACCTTATGGGACTTTCCTACTTGGCAGTACATCTACGTATTA
GTCATCGCTATTACCATGGTGATGCGGTTTTGGCAGTACATCAATGGGCGTGGATAGCGGTTTGACTCACGGGGATTTCCAAGTCTCCACCCCATTG
ACGTCAATGGGAGTTTGTTTTGGCACCAAAATCAACGGGACTTTCCAAAATGTCGTAACAACTCCGCCCCATTGACGCAAATGGGCGGTAGGCGTGT
ACGGTGGGAGGTCTATATAAGCAGAGCTCTCTGGCTAACTAGAGAACCCACTGCTTACTGGCTTATCGAAATTAATACGACTCACTATAGGGAGACC
CAAGCTGGCTAGCGTTTAAACTTAAGCTTGCCCGGGATCCACCGGTCGCCACCATGGTGAGCAAGGGCGAGGAGCTGTTCACCGGGGTGGTGCCCAT
CCTGGTCGAGCTGGACGGCGACGTAAACGGCCACAAGTTCAGCGTGTCCGGCGAGGGCGAGGGCGATGCCACCTACGGCAAGCTGACCCTGAAGTTC
ATCTGCACCACCGGCAAGCTGCCCGTGCCCTGGCCCACCCTCGTGACCACCCTGACCTACGGCGTGCAGTGCTTCAGCCGCTACCCCGACCACATGA
AGCAGCACGACTTCTTCAAGTCCGCCATGCCCGAAGGCTACGTCCAGGAGCGCACCATCTTCTTCAAGGACGACGGCAACTACAAGACCCGCGCCGA
GGTGAAGTTCGAGGGCGACACCCTGGTGAACCGCATCGAGCTGAAGGGCATCGACTTCAAGGAGGACGGCAACATCCTGGGGCACAAGCTGGAGTAC
AACTACAACAGCCACAACGTCTATATCATGGCCGACAAGCAGAAGAACGGCATCAAGGTGAACTTCAAGATCCGCCACAACATCGAGGACGGCAGCG
TGCAGCTCGCCGACCACTACCAGCAGAACACCCCCATCGGCGACGGCCCCGTGCTGCTGCCCGACAACCACTACCTGAGCACCCAGTCCGCCCTGAG
CAAAGACCCCAACGAGAAGCGCGATCACATGGTCCTGCTGGAGTTCGTGACCGCCGCCGGGATCACTCTCGGCATGGACGAGCTGTACAAGTAAGGC
CGCGACTCTAGAGGGCCCGTTTAAACCCGCTGATCAGCCTCGACTGTGCCTTCTAGTTGCCAGCCATCTGTTGTTTGCCCCTCCCCCGTGCCTTCCT
TGACCCTGGAAGGTGCCACTCCCACTGTCCTTTCCTAATAAAATGAGGAAATTGCATCGCATTGTCTGAGTAGGTGTCATTCTATTCTGGGGGGTGG
GGTGGGGCAGGACAGCAAGGGGGAGGATTGGGAAGACAATAGCAGGCATGCTGGGGATGCGGTGGGCTCTATGGCTTCTGAGGCGGAAAGAACCAGC
TGGGGCTCTAGGGGGTATCCCCACGCGCCCTGTAGCGGCGCATTAAGCGCGGCGGGTGTGGTGGTTACGCGCAGCGTGACCGCTACACTTGCCAGCG
CCCTAGCGCCCGCTCCTTTCGCTTTCTTCCCTTCCTTTCTCGCCACGTTCGCCGGCTTTCCCCGTCAAGCTCTAAATCGGGGGCTCCCTTTAGGGTT
CCGATTTAGTGCTTTACGGCACCTCGACCCCAAAAAACTTGATTAGGGTGATGGTTCACGTAGTGGGCCATCGCCCTGATAGACGGTTTTTCGCCCT
TTGACGTTGGAGTCCACGTTCTTTAATAGTGGACTCTTGTTCCAAACTGGAACAACACTCAACCCTATCTCGGTCTATTCTTTTGATTTATAAGGGA
TTTTGCCGATTTCGGCCTATTGGTTAAAAAATGAGCTGATTTAACAAAAATTTAACGCGAATTAATTCTGTGGAATGTGTGTCAGTTAGGGTGTGGA
AAGTCCCCAGGCTCCCCAGCAGGCAGAAGTATGCAAAGCATGCATCTCAATTAGTCAGCAACCAGGTGTGGAAAGTCCCCAGGCTCCCCAGCAGGCA
GAAGTATGCAAAGCATGCATCTCAATTAGTCAGCAACCATAGTCCCGCCCCTAACTCCGCCCATCCCGCCCCTAACTCCGCCCAGTTCCGCCCATTC
TCCGCCCCATGGCTGACTAATTTTTTTTATTTATGCAGAGGCCGAGGCCGCCTCTGCCTCTGAGCTATTCCAGAAGTAGTGAGGAGGCTTTTTTGGA
GGCCTAGGCTTTTGCAAAAAGCTCCCGGGAGCTTGTATATCCATTTTCGGATCTGATCAAGAGACAGGATGAGGATCGTTTCGCATGATTGAACAAG
ATGGATTGCACGCAGGTTCTCCGGCCGCTTGGGTGGAGAGGCTATTCGGCTATGACTGGGCACAACAGACAATCGGCTGCTCTGATGCCGCCGTGTT
CCGGCTGTCAGCGCAGGGGCGCCCGGTTCTTTTTGTCAAGACCGACCTGTCCGGTGCCCTGAATGAACTGCAGGACGAGGCAGCGCGGCTATCGTGG
CTGGCCACGACGGGCGTTCCTTGCGCAGCTGTGCTCGACGTTGTCACTGAAGCGGGAAGGGACTGGCTGCTATTGGGCGAAGTGCCGGGGCAGGATC
TCCTGTCATCTCACCTTGCTCCTGCCGAGAAAGTATCCATCATGGCTGATGCAATGCGGCGGCTGCATACGCTTGATCCGGCTACCTGCCCATTCGA
CCACCAAGCGAAACATCGCATCGAGCGAGCACGTACTCGGATGGAAGCCGGTCTTGTCGATCAGGATGATCTGGACGAAGAGCATCAGGGGCTCGCG
CCAGCCGAACTGTTCGCCAGGCTCAAGGCGCGCATGCCCGACGGCGAGGATCTCGTCGTGACCCATGGCGATGCCTGCTTGCCGAATATCATGGTGG
AAAATGGCCGCTTTTCTGGATTCATCGACTGTGGCCGGCTGGGTGTGGCGGACCGCTATCAGGACATAGCGTTGGCTACCCGTGATATTGCTGAAGA
GCTTGGCGGCGAATGGGCTGACCGCTTCCTCGTGCTTTACGGTATCGCCGCTCCCGATTCGCAGCGCATCGCCTTCTATCGCCTTCTTGACGAGTTC
TTCTGAGCGGGACTCTGGGGTTCGAAATGACCGACCAAGCGACGCCCAACCTGCCATCACGAGATTTCGATTCCACCGCCGCCTTCTATGAAAGGTT
GGGCTTCGGAATCGTTTTCCGGGACGCCGGCTGGATGATCCTCCAGCGCGGGGATCTCATGCTGGAGTTCTTCGCCCACCCCAACTTGTTTATTGCA
GCTTATAATGGTTACAAATAAAGCAATAGCATCACAAATTTCACAAATAAAGCATTTTTTTCACTGCATTCTAGTTGTGGTTTGTCCAAACTCATCA
ATGTATCTTATCATGTCTGTATACCGTCGACCTCTAGCTAGAGCTTGGCGTAATCATGGTCATAGCTGTTTCCTGTGTGAAATTGTTATCCGCTCAC
AATTCCACACAACATACGAGCCGGAAGCATAAAGTGTAAAGCCTGGGGTGCCTAATGAGTGAGCTAACTCACATTAATTGCGTTGCGCTCACTGCCC
GCTTTCCAGTCGGGAAACCTGTCGTGCCAGCTGCATTAATGAATCGGCCAACGCGCGGGGAGAGGCGGTTTGCGTATTGGGCGCTCTTCCGCTTCCT
CGCTCACTGACTCGCTGCGCTCGGTCGTTCGGCTGCGGCGAGCGGTATCAGCTCACTCAAAGGCGGTAATACGGTTATCCACAGAATCAGGGGATAA
CGCAGGAAAGAACATGTGAGCAAAAGGCCAGCAAAAGGCCAGGAACCGTAAAAAGGCCGCGTTGCTGGCGTTTTTCCATAGGCTCCGCCCCCCTGAC
GAGCATCACAAAAATCGACGCTCAAGTCAGAGGTGGCGAAACCCGACAGGACTATAAAGATACCAGGCGTTTCCCCCTGGAAGCTCCCTCGTGCGCT
CTCCTGTTCCGACCCTGCCGCTTACCGGATACCTGTCCGCCTTTCTCCCTTCGGGAAGCGTGGCGCTTTCTCATAGCTCACGCTGTAGGTATCTCAG
TTCGGTGTAGGTCGTTCGCTCCAAGCTGGGCTGTGTGCACGAACCCCCCGTTCAGCCCGACCGCTGCGCCTTATCCGGTAACTATCGTCTTGAGTCC
AACCCGGTAAGACACGACTTATCGCCACTGGCAGCAGCCACTGGTAACAGGATTAGCAGAGCGAGGTATGTAGGCGGTGCTACAGAGTTCTTGAAGT
GGTGGCCTAACTACGGCTACACTAGAAGAACAGTATTTGGTATCTGCGCTCTGCTGAAGCCAGTTACCTTCGGAAAAAGAGTTGGTAGCTCTTGATC
CGGCAAACAAACCACCGCTGGTAGCGGTTTTTTTGTTTGCAAGCAGCAGATTACGCGCAGAAAAAAAGGATCTCAAGAAGATCCTTTGATCTTTTCT
ACGGGGTCTGACGCTCAGTGGAACGAAAACTCACGTTAAGGGATTTTGGTCATGAGATTATCAAAAAGGATCTTCACCTAGATCCTTTTAAATTAAA
AATGAAGTTTTAAATCAATCTAAAGTATATATGAGTAAACTTGGTCTGACAGTTACCAATGCTTAATCAGTGAGGCACCTATCTCAGCGATCTGTCT
ATTTCGTTCATCCATAGTTGCCTGACTCCCCGTCGTGTAGATAACTACGATACGGGAGGGCTTACCATCTGGCCCCAGTGCTGCAATGATACCGCGA
GACCCACGCTCACCGGCTCCAGATTTATCAGCAATAAACCAGCCAGCCGGAAGGGCCGAGCGCAGAAGTGGTCCTGCAACTTTATCCGCCTCCATCC
AGTCTATTAATTGTTGCCGGGAAGCTAGAGTAAGTAGTTCGCCAGTTAATAGTTTGCGCAACGTTGTTGCCATTGCTACAGGCATCGTGGTGTCACG
CTCGTCGTTTGGTATGGCTTCATTCAGCTCCGGTTCCCAACGATCAAGGCGAGTTACATGATCCCCCATGTTGTGCAAAAAAGCGGTTAGCTCCTTC
GGTCCTCCGATCGTTGTCAGAAGTAAGTTGGCCGCAGTGTTATCACTCATGGTTATGGCAGCACTGCATAATTCTCTTACTGTCATGCCATCCGTAA
GATGCTTTTCTGTGACTGGTGAGTACTCAACCAAGTCATTCTGAGAATAGTGTATGCGGCGACCGAGTTGCTCTTGCCCGGCGTCAATACGGGATAA
TACCGCGCCACATAGCAGAACTTTAAAAGTGCTCATCATTGGAAAACGTTCTTCGGGGCGAAAACTCTCAAGGATCTTACCGCTGTTGAGATCCAGT
TCGATGTAACCCACTCGTGCACCCAACTGATCTTCAGCATCTTTTACTTTCACCAGCGTTTCTGGGTGAGCAAAAACAGGAAGGCAAAATGCC
```

The three G-quadruplex containing plasmids were then generated by inserting the following sequences between the **XbaI** and **ApaI** sites of **pcDNA3.1(+) eGFP**.

pcDNA3.1(+) eGFP PPP1R8 G4

TGTGGACTTGACTCCTGTTGTGCCGTCAGCAGTGAACATGAACCCTGCACCAAACCCTGCAGTCTATAACCCTGAAGCTGTAAATGAACCCAAGAAG
AAGAAATATGCAAAAGAGGCTTGGCCAGGCAAGAAGCCCACACCTTCCTTGCTGATTTGATATTTTTGGTCATGGAGAAGGGTGGGATTGGGTGGGA
ATGGGGTGGAAGGGTGATGGGGAGCTAATGAACTAGGGAGAAAAACTTTCCATGTGTGCGGTATCGTCTTTCAGAATGTCTCCTGGCATCCTAACCA
TGTAATATGACAATTGGGGGTGGG

pcDNA3.1(+) eGFP SH3BP5 G4

AGGCTACCATGAGCTGCACTTTTTGGGGTGGGAAAGGTGAATGCCAGTGGGGATGCGGGGGGATGAGGGTAGGAGGGACTTATAGAAGGGGATTTGT
GGCTGTGGGGGAGAAGGTTCTACAGCATAAGCCTTATCCTGCCAGCCAAGGGGATTTATTCTAAGAG

pcDNA3.1(+) eGFP EML2 G4

TGACCACAGGGGGCAAGGACACCAGTGTGCTACAGTGGCGGGTGGTCTGATGCGGCCAGGGAAGAGTCAGGTGTCAGGGCAGGAATTCTATTTTCGG
GAGATGTCTATTGCCGAGTAGAGTAATATATACCCAGAGTATGTCTATAGCAGAGGGGGTTATGGGGGCGGGAGGGTAGACTGACATACAGAAGTCT
CTATTTATCCGGGTGGGAAGAGGGAGTCACATCGCTTTGGGGATCCATTGGTGTTTGGTTTGGGGTGTTTTTTAAG

Supplementary Figure S1

A

Sample	Total reads	Mapped reads	Uniquely mapped reads
Rep 1 Control	76,857,781	70,391,346 (91.6%)	33,073,744 (47.0%)
Rep 1 RNase R	68,321,830	60,172,140 (88.1%)	41,979,262 (69.8%)
Rep 2 Control	68,408,807	60,804,034 (88.9%)	55,214,051 (91.8%)
Rep 2 RNase R	77,527,572	67,793,949 (87.4%)	53,997,296 (79.6%)

B

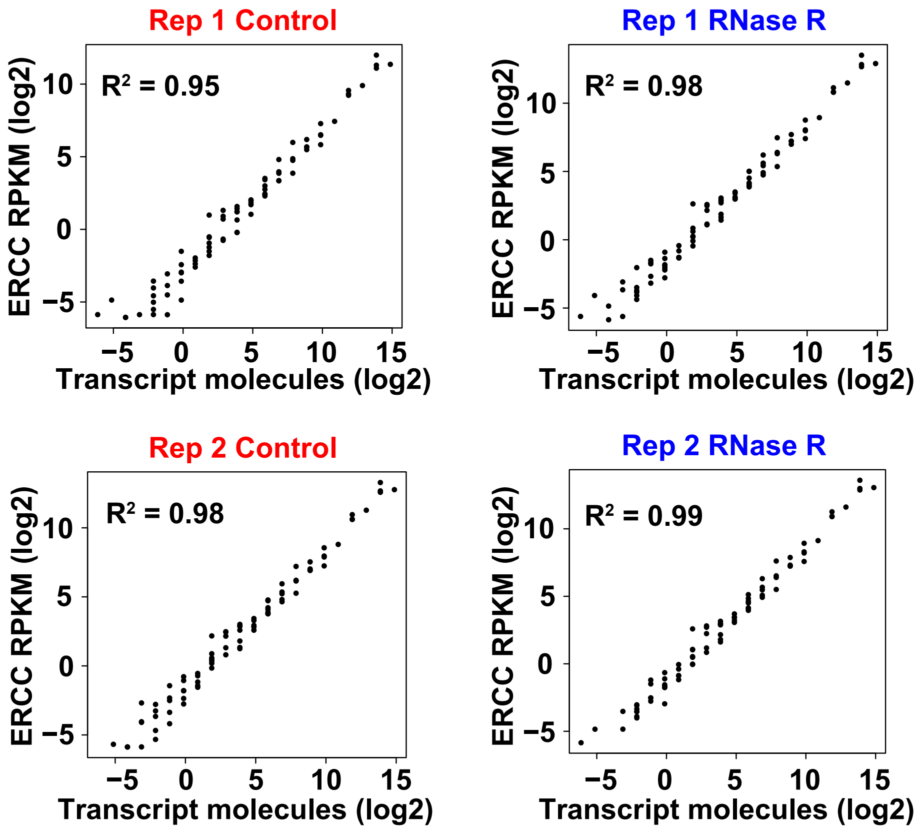

C

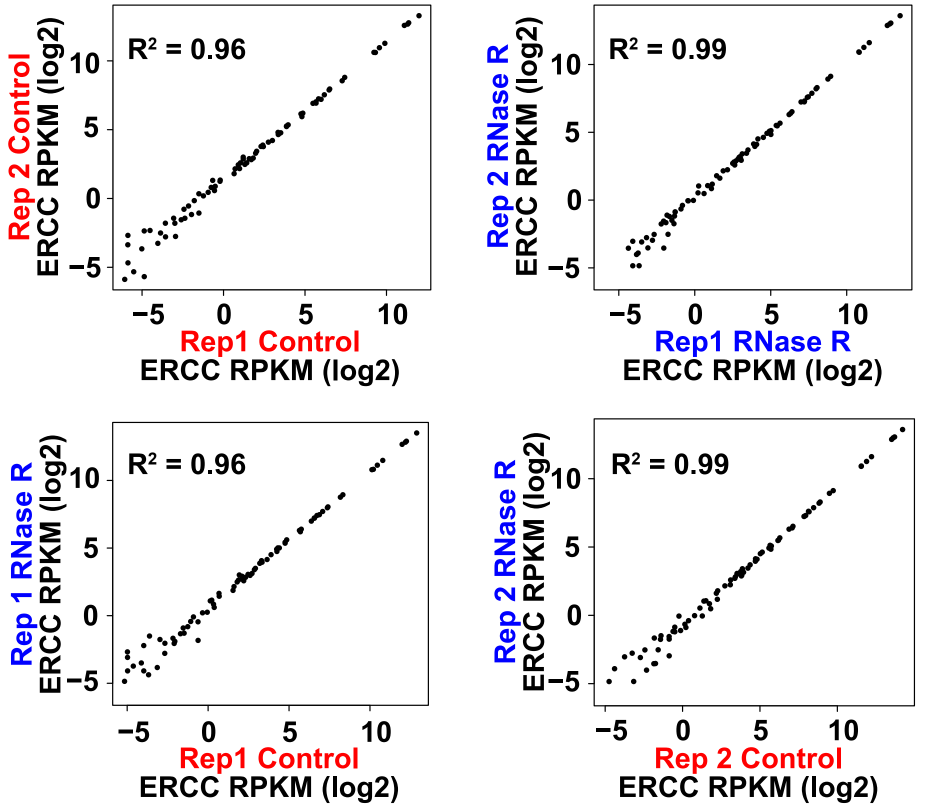

Supplementary Figure S2

A

No. of predicted circRNAs

Sample	CIRI2	CIRCexplorer2	Both Algorithms
Rep 1 Control	1794	1282	1034
Rep 1 RNase R	10892	7801	6670
Rep 2 Control	4776	3305	2891
Rep 2 RNase R	15531	10673	9533

B

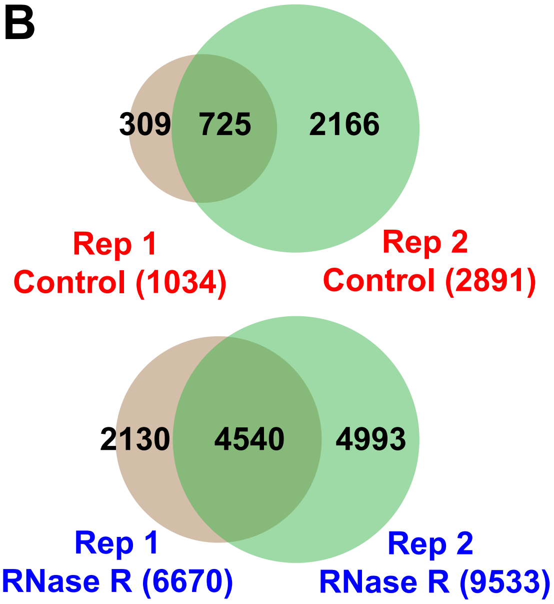

C

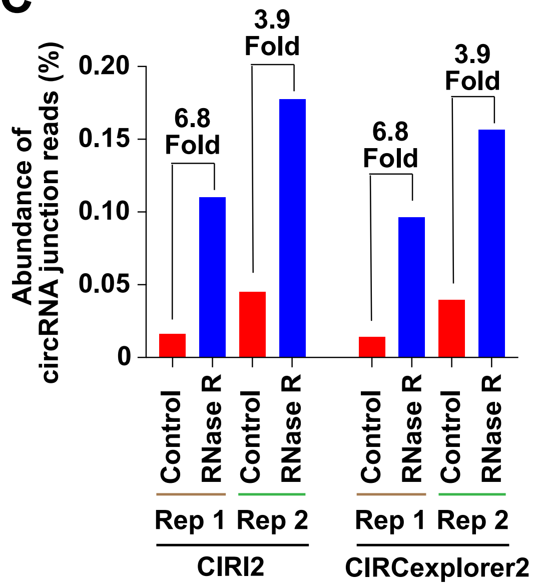

D

Sample	No. of predicted ciRNAs (CIRCexplorer2)
Rep 1 Control	17
Rep 1 RNase R	464
Rep 2 Control	43
Rep 2 RNase R	581

E

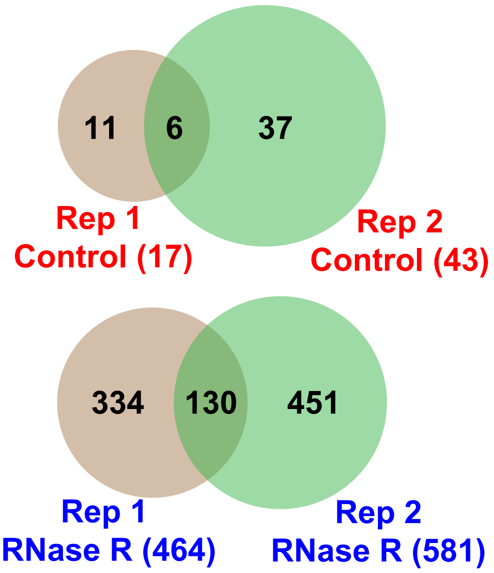

F

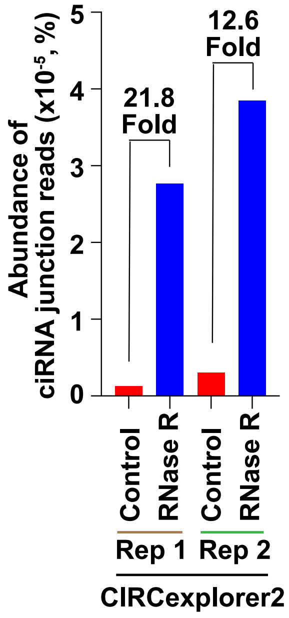

G

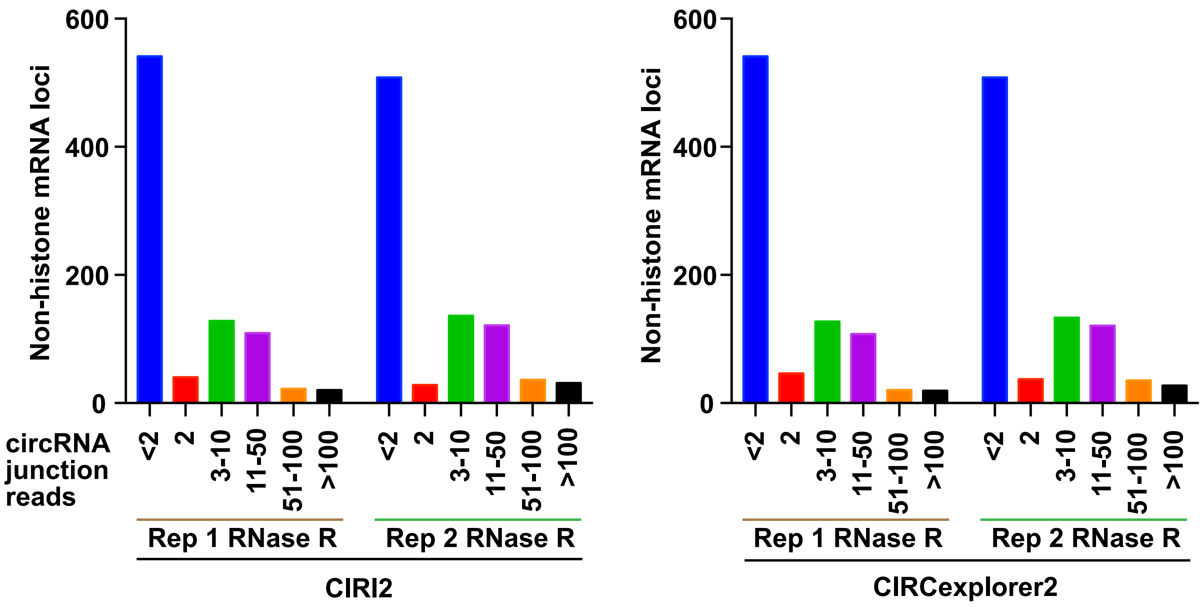

Supplementary Figure S3

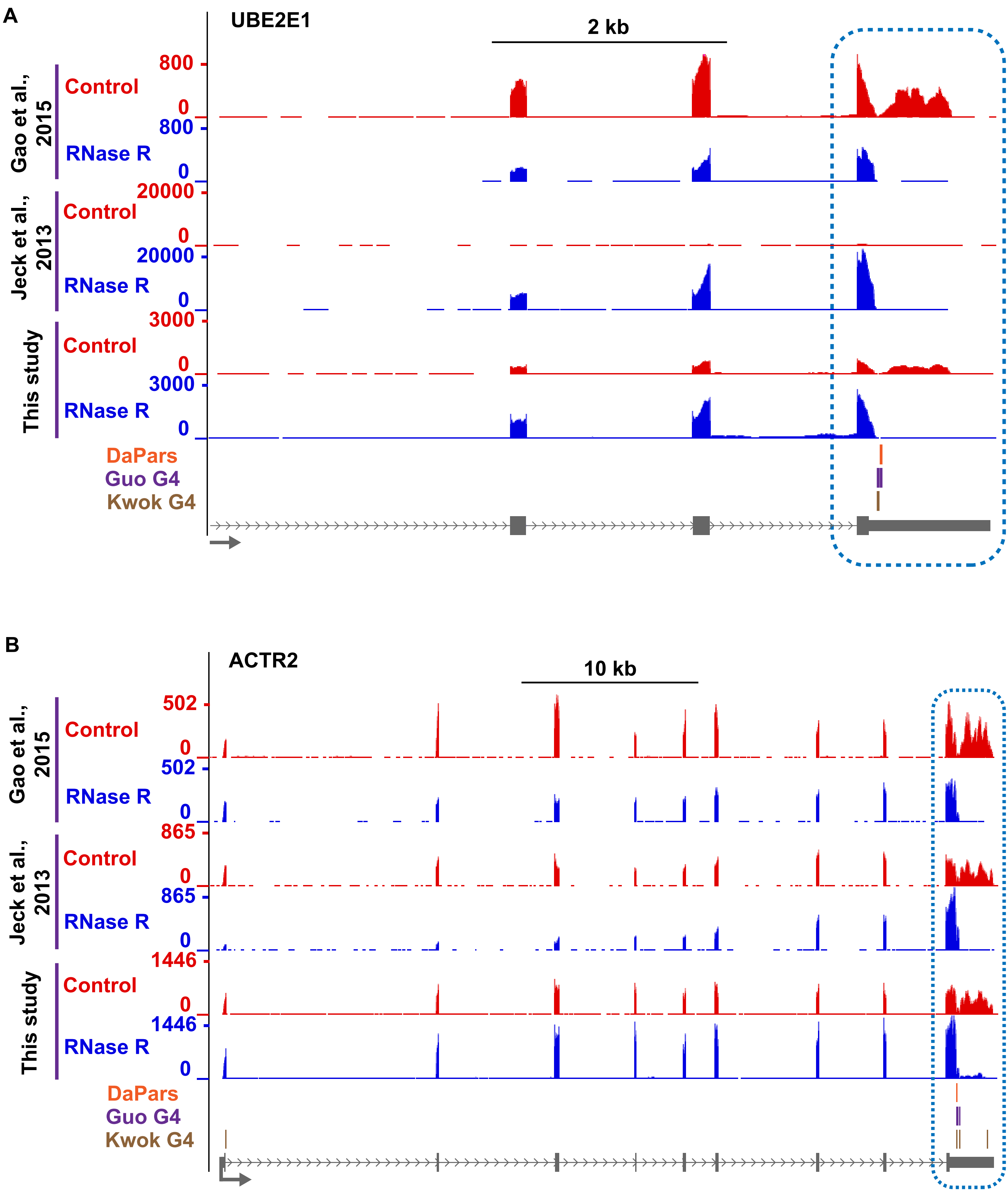

Supplementary Figure S4

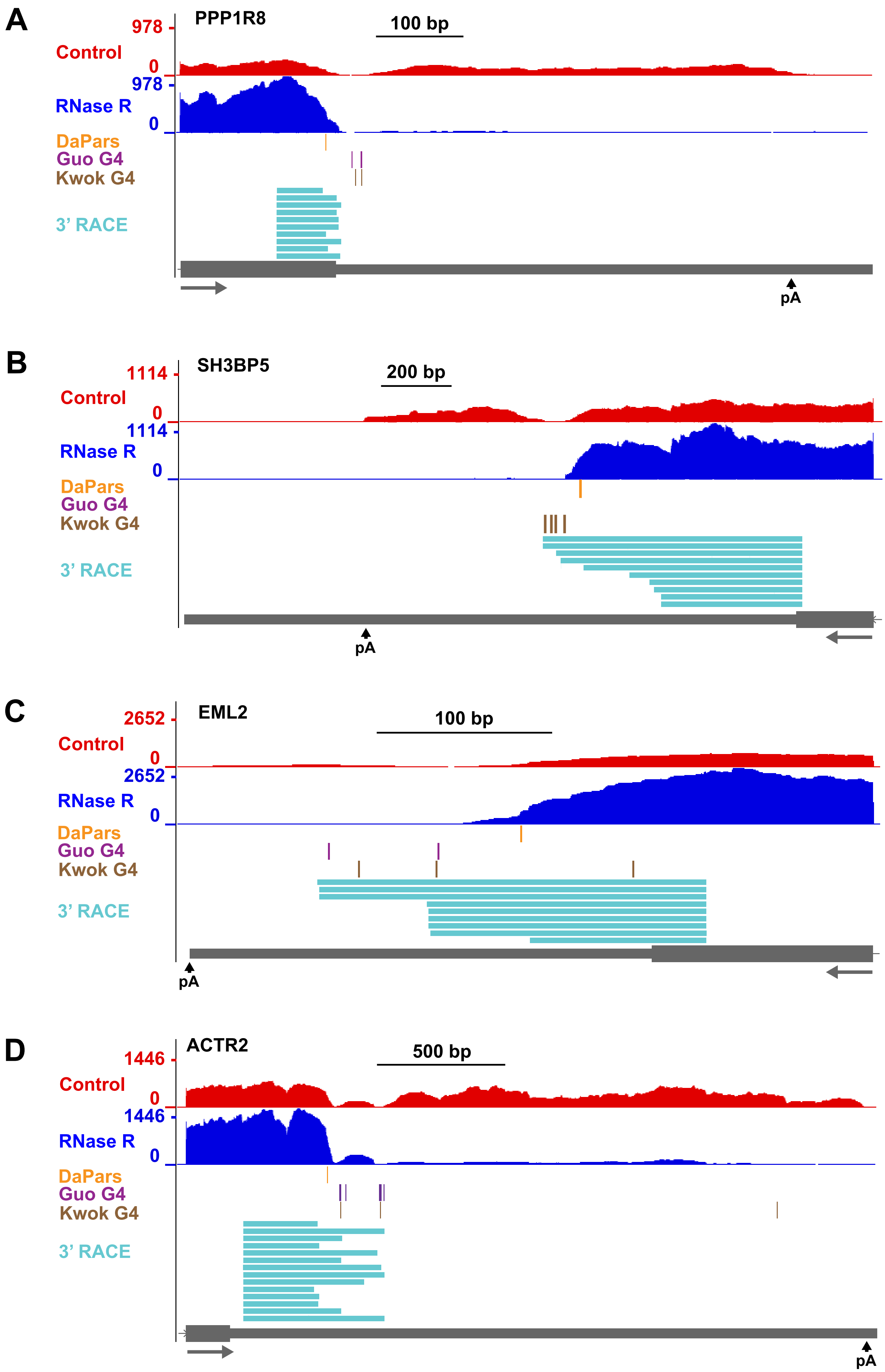

Supplementary Figure S5

	with Guo G4	without Guo G4
Stalling sites (337)	212	125
Controls (33,700)	1,014	32,686

Fisher's Exact Test; *P*-value < 2.2e-16

	with Kwok G4	without Kwok G4
Stalling sites (337)	227	110
Controls (33,700)	1,567	32,133

Fisher's Exact Test; *P*-value < 2.2e-16

Supplementary Figure S6

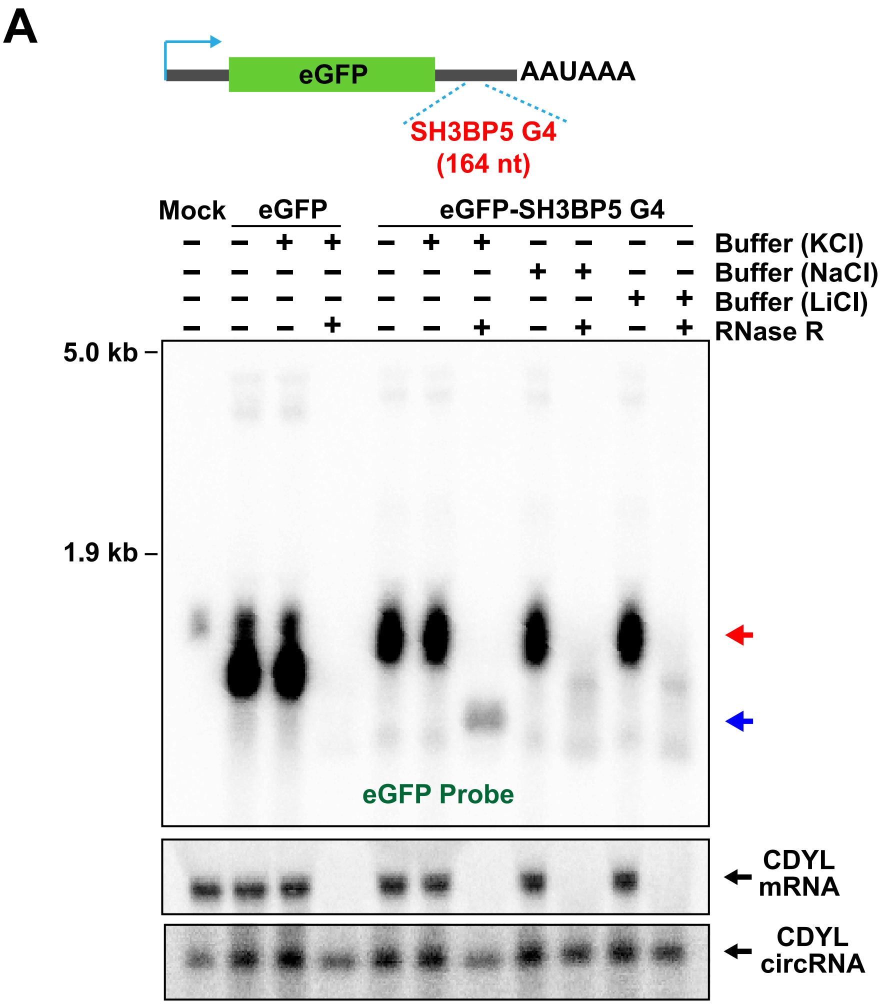

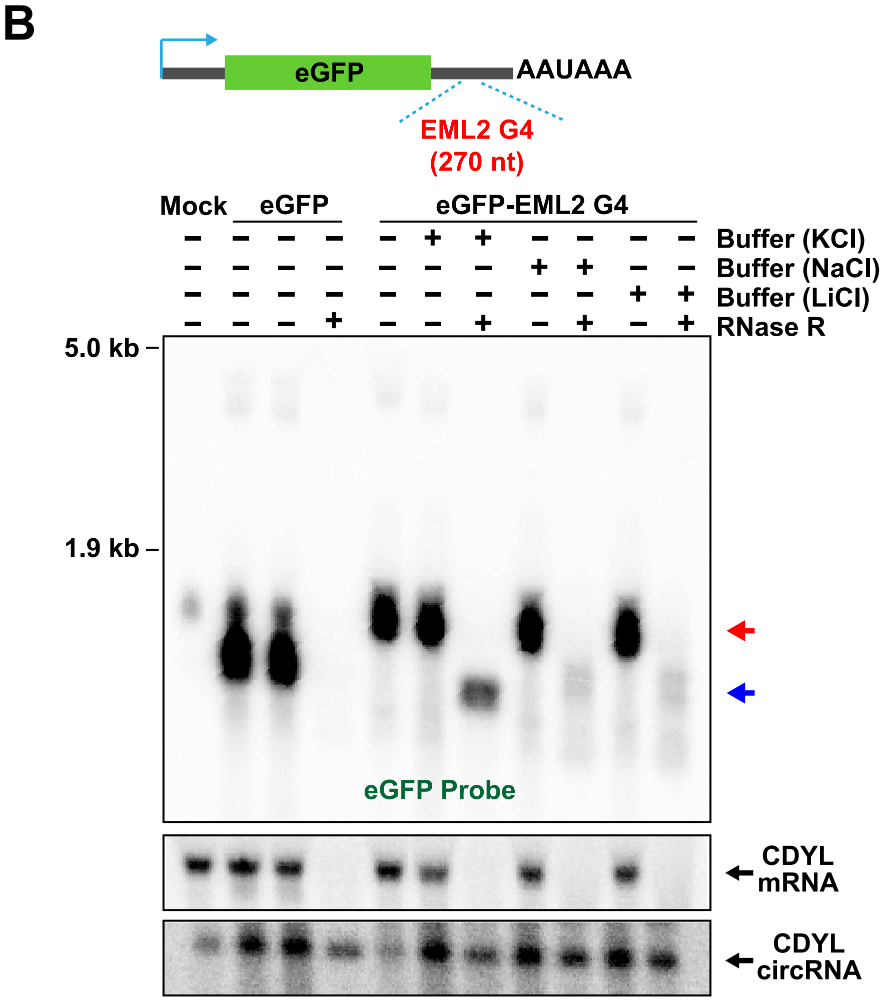

Supplementary Figure S7

A

Group	Sample	Total reads	Mapped reads	Uniquely mapped reads
Control (LiCl)	Rep 1	70,248,650	63,983,339 (91.1%)	58,687,452 (91.2%)
Control (LiCl)	Rep 2	43,910,890	39,998,941 (91.1%)	36,896,272 (92.2%)
Control (LiCl)	Rep 3	58,697,383	53,405,357 (91.0%)	49,094,965 (91.9%)
A-Tailing + RNase R (LiCl)	Rep 1	24,593,985	21,221,362 (86.3%)	13,085,924 (61.7%)
A-Tailing + RNase R (LiCl)	Rep 2	23,230,377	20,029,202 (86.2%)	12,192,692 (60.9%)
A-Tailing + RNase R (LiCl)	Rep 3	32,621,247	28,433,685 (87.2%)	17,665,257 (62.1%)

B

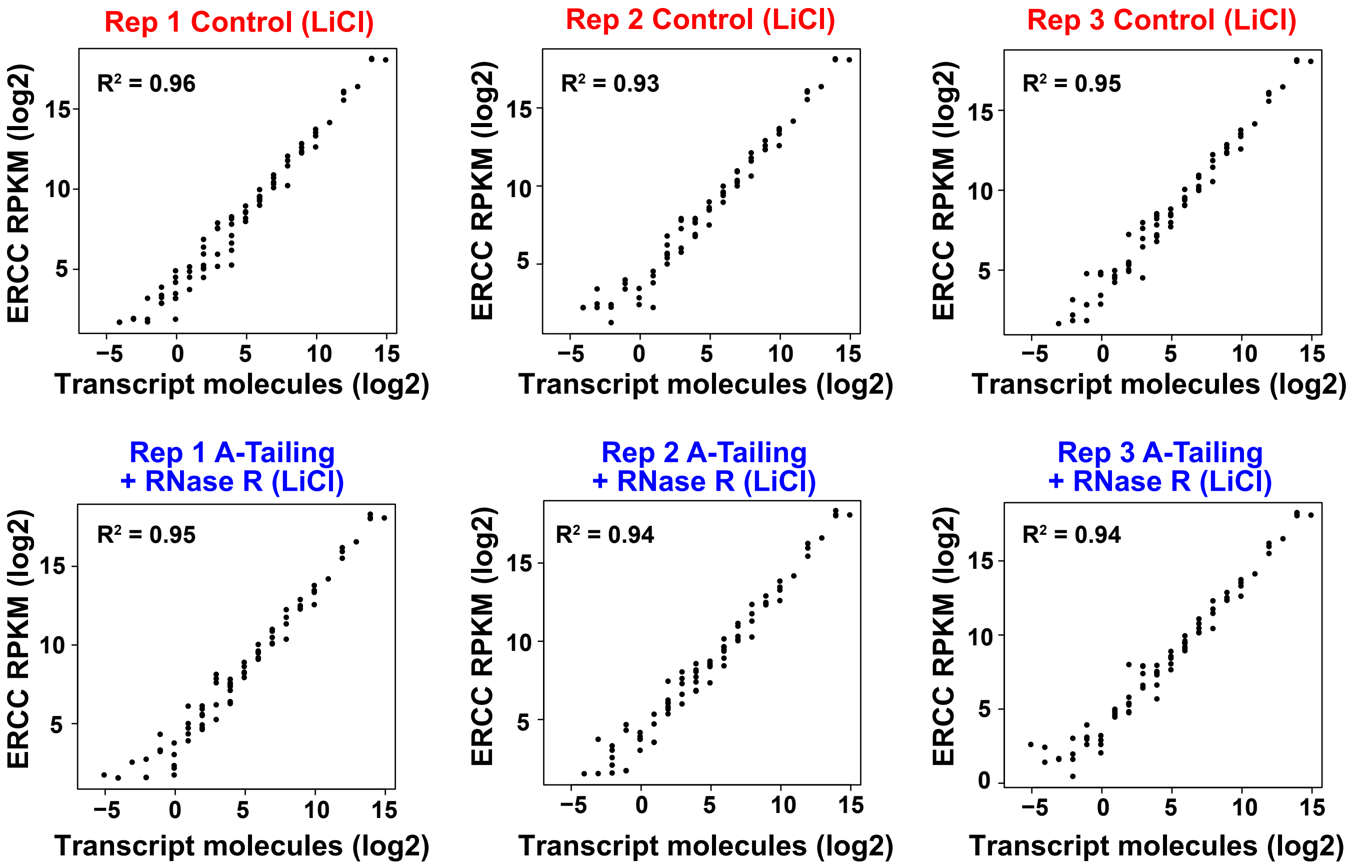

Supplementary Figure S8

A

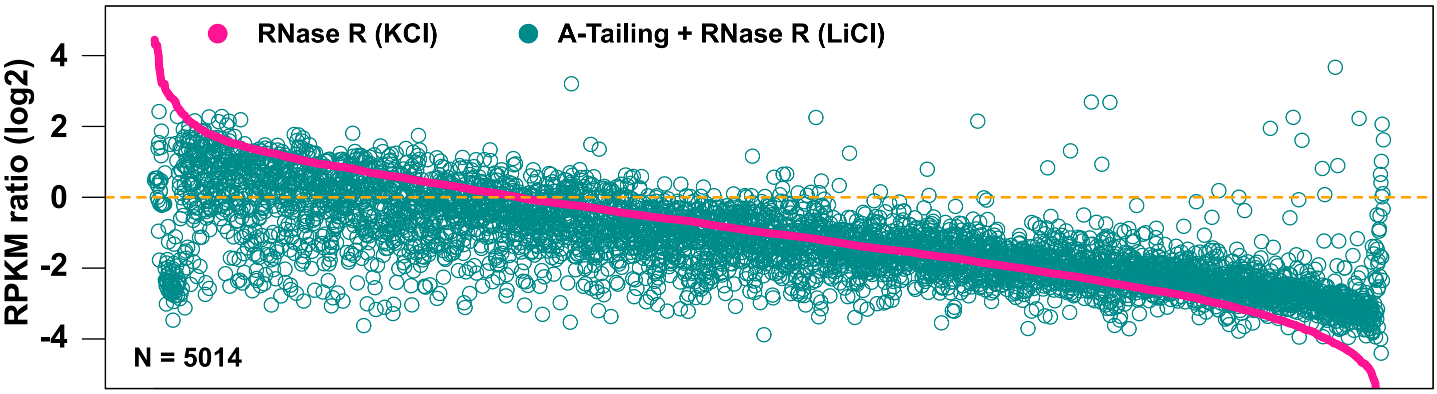

B

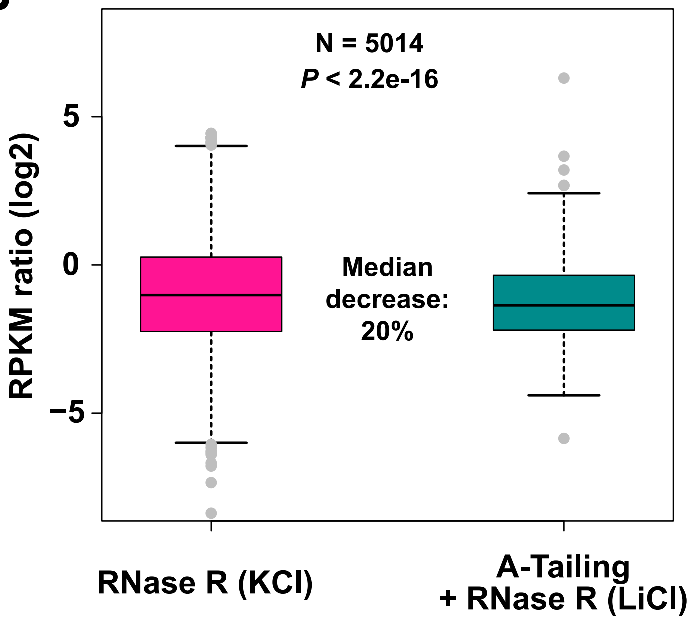

Supplementary Figure S9

A

No. of predicted circRNAs

	Sample	CIRI2	CIRCexplorer2	Both algorithms
Control (LiCl)	Rep 1	5129	3396	3091
	Rep 2	3371	2225	2053
	Rep 3	4661	3103	2823
A-Tailing + RNase R (LiCl)	Rep 1	11171	7395	6848
	Rep 2	10599	6984	6470
	Rep 3	13022	8556	7979

B

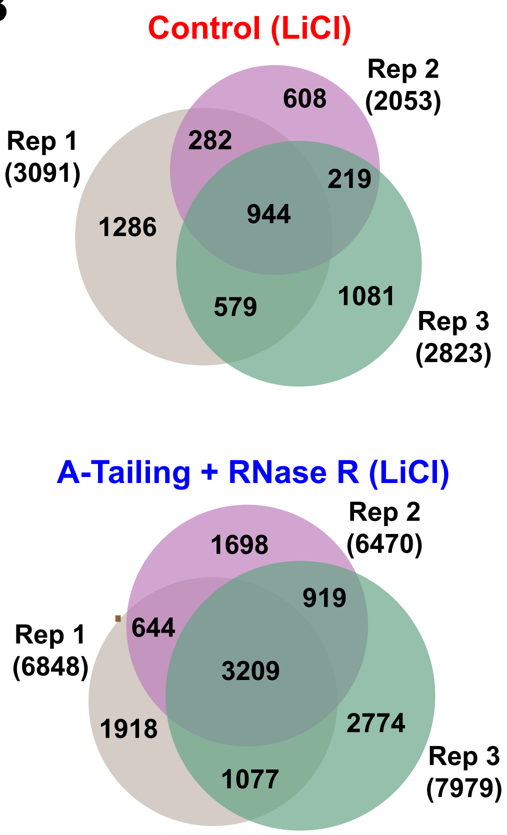

C

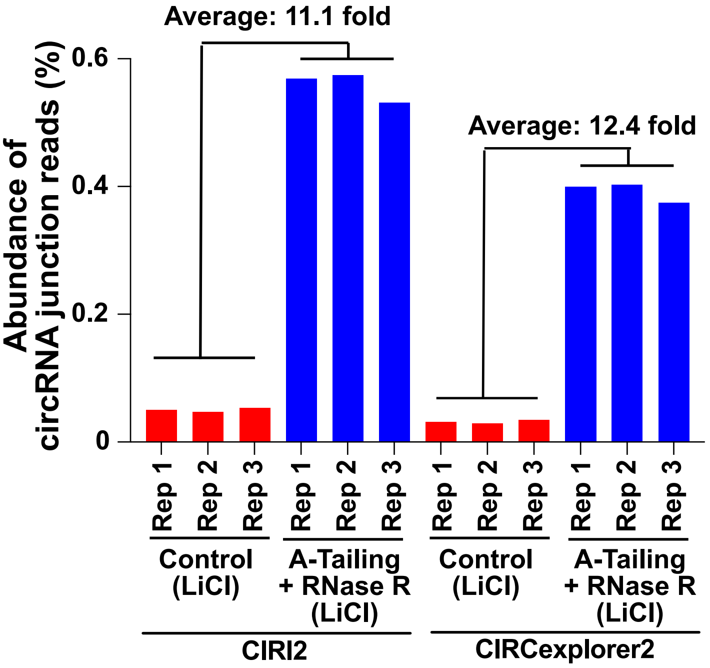

D

	Sample	No. of predicted ciRNAs (CIRCexplorer2)
Control (LiCl)	Rep 1	22
	Rep 2	11
	Rep 3	20
A-Tailing + RNase R (LiCl)	Rep 1	113
	Rep 2	109
	Rep 3	107

E

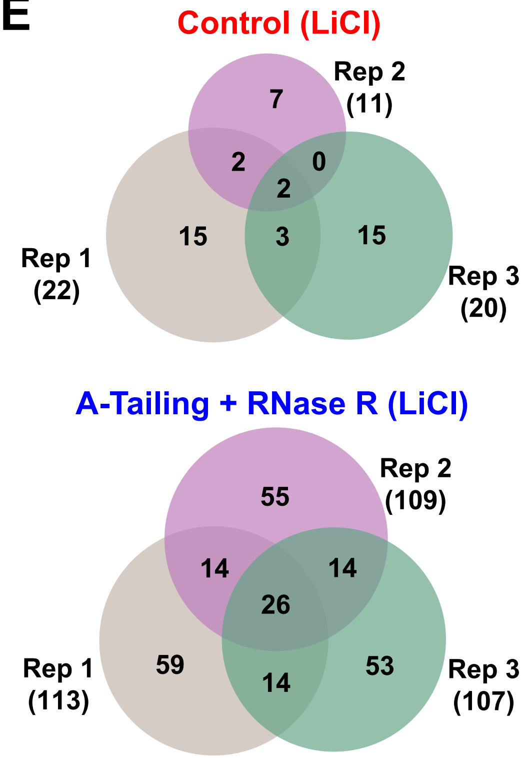

F

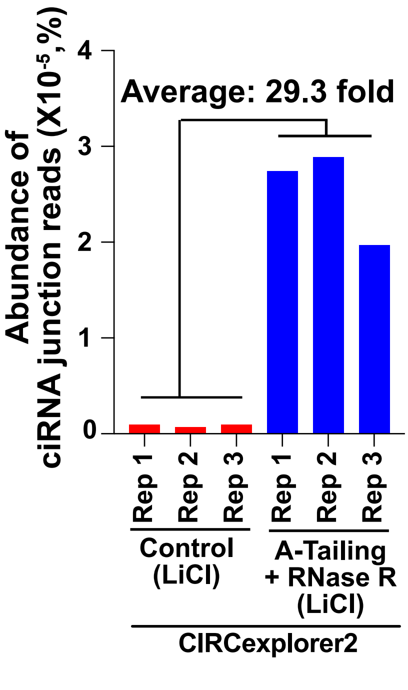

Supplement: gkz576_Supplemental_Files [file gkz576_supplemental_files.zip › Supplementary Material.pdf]
